# Greywater recycling and solar photovoltaic integration for sustainable water and energy management in urban Egypt

**DOI:** 10.1038/s41598-026-49932-y

**Published:** 2026-05-06

**Authors:** Ahmed Abdo, Ahmed M. Othman, Dalia Ahmed

**Affiliations:** 1 Environmental Engineering Department, Faculty of Engineering, Zagazig University, Zagazig, 44519 Egypt; 2 Electrical Engineering Department, Faculty of Engineering, Zagazig University, Zagazig, 44519 Egypt

**Keywords:** Greywater reuse, Solar energy, Sustainable urban development, Energy and society, Energy science and technology, Engineering, Environmental sciences, Environmental social sciences

## Abstract

This study evaluates an integrated greywater reuse and rooftop solar photovoltaic (PV) concept for a luxury residential compound in New Cairo, Egypt (365 buildings; 7,512 units). Based on stated per‑capita end‑use assumptions, total greywater production is 6,300 m^3^/day (2,299,500 m^3^/year), while toilet flushing demand is 2,700 m^3^/day (985,500 m^3^/year). A physico‑chemical onsite treatment train (coagulation, multimedia filtration, chlorination) is specified at 6,300 m^3^/day; using a 5% filter backwash assumption, annual hydraulic recovery is 95% (2,184,525 m^3^/year). The recovered volume exceeds the flushing demand, indicating demand‑limited potable‑water offset for the quantified end use and potential surplus for additional non‑potable applications. The greywater system CAPEX is 3.37 million USD (535 USD/(m^3^/day)) with disaggregated components for in‑building supply, transfer networks, and the treatment plant. Total OPEX is 0.06 USD/m^3^ (137,970 USD/year at design flow), yielding a straight‑line levelized cost of water (LCOW) of 0.133 USD/m^3^ on a treated‑volume basis and 0.311 USD/m^3^ on a potable‑offset basis over the 20‑year analysis horizon. For the PV system, the total initial cost for 365 buildings is 666,455,000 EGP, with modeled annual electricity cost savings of 5,348,550 EGP and an indicative simple payback of approximately 10 years. Overall, the results quantify the magnitude of potable‑water offset and cost metrics achievable at compound scale.

**Supplementary Information:**

The online version contains supplementary material available at 10.1038/s41598-026-49932-y.

## Introduction

The growing human population continues to place increasing pressure on natural resources—particularly clean water—required for domestic, agricultural, and industrial use. Without reliable and sustainable water sources, many risk facing severe shortages and a rise in diseases linked to polluted water^1^. In light of these challenges, wastewater treatment has gained global attention not only for its economic advantages but also for its critical role in environmental sustainability. It is now widely recognized as a key strategy for preserving water quality and promoting efficient resource use, ensuring that water is not wasted, polluted, or mismanaged^2^.

Amid escalating global water scarcity, the reuse of treated wastewater—particularly greywater from household activities—has emerged as a viable and increasingly necessary solution. In many developing countries, blackwater is directed to septic tanks, while greywater is often discharged untreated into the environment, especially in rural areas with limited infrastructure. Even in urban settings, centralized wastewater treatment plants are usually designed to handle combined flows without separating greywater from blackwater. Given that greywater is of lower quality than potable water but significantly cleaner than sewage, it can be treated through relatively simple, decentralized systems, offering both practicality and efficiency^3^. For example, in Saudi Arabia, large quantities of ablution water—a form of greywater—are now being recognized as a recoverable and reusable resource^4^.

Greywater’s relatively low organic and microbial load makes it especially suitable for decentralized reuse. When used for non-potable applications such as toilet flushing and irrigation, it can significantly reduce pressure on freshwater sources while mitigating the environmental burden of conventional wastewater discharge. In recent years, innovative treatment technologies have expanded the feasibility and safety of greywater recycling, reinforcing its role in sustainable, water-efficient systems^5^.

Agriculture, power plants, and industrial facilities in Egypt discharge approximately 13.5, 4.2, and 0.15 billion m^3^/year of untreated wastewater each year into the Nile River—Egypt’s primary source of freshwater^6^. This widespread pollution accounts for nearly 29% of the nation’s annual freshwater loss, significantly constraining the amount available for domestic use^7^. In response to this escalating threat, greywater management has gained traction as one of the most promising non-conventional water sources, offering a practical and scalable solution to support Egypt’s water security and reduce dependency on vulnerable freshwater reserves.

By 2050, Egypt’s renewable water supply gap is expected to more than double. Addressing this gap will require significant technical effort and financial investment. Policymakers are responding by prioritizing water demand management, particularly within agriculture, which consumes the majority of national water resources. Once Egypt reaches the limits of its renewable freshwater resources, it will be compelled to rely on costly alternatives such as seawater desalination, brackish water treatment, and advanced wastewater reuse. This looming shift underscores the urgency of implementing water conservation measures as a national priority^8^. Greywater reuse stands out as a cost-effective and scalable strategy, with the potential to contribute 4.15–8.30 billion m^3^/year to Egypt’s water supply^9^. Integrating greywater systems into large-scale urban and residential developments can significantly enhance national water resilience. Moreover, this approach supports broader objectives outlined in the 2030 Agenda for Sustainable Development, which link improved water access with reduced inequality^6^.

With the growing focus on renewable and clean energy resources, hybrid solar systems have become increasingly significant. Solar photovoltaic (PV) energy offers a reliable and sustainable power source, with its output naturally varying according to daily sunlight availability and weather conditions. By integrating PV systems with energy storage solutions, these fluctuations can be effectively managed, ensuring a continuous and dependable electricity supply for various applications. Solar PVs have emerged as a significant solution to meet the rising demand for electricity, especially in remote areas were traditional power transmission results in substantial energy losses. Additionally, the dramatic increase in load demand is often unmatched by investments in distribution and transmission systems^10,11^.

Physicochemical treatment trains — combining coagulation, multimedia filtration, and chlorination — have been widely reported as the dominant choice for onsite greywater reuse due to their robust suspended solids removal and relatively simple operation compared with biological alternatives^12^. Noutsopoulos et al.^13^ demonstrated that such treatment trains reliably achieve effluent quality suitable for non-potable reuse applications, with BOD_5_ and TSS consistently below 10 mg/L. Jefferson et al.^14^ further established that the organic content and chlorine demand of greywater are the primary treatment design parameters, directly informing disinfection dosing requirements. Regarding economics, Friedler and Hadari^15^ showed that onsite greywater systems in multi-store buildings become economically viable when serving a sufficiently large population, as shared infrastructure spreads fixed capital costs — a finding directly applicable to the compound-scale system examined in this study. Evidence from water reuse facilities across the Middle East and North Africa reports OPEX for tertiary treatment in the range of 0.03–0.09 USD/m^3^^16^, consistent with the independently reported range of 0.069–0.154 EUR/m^3^ for Mediterranean reuse systems^17^, providing benchmarks against which the financial outputs of this study are directly compared.

Studies integrating greywater reuse with solar PV have generally operated at small scale and with a single coupling objective. Waris and Ghaith^18^ designed a PV-powered greywater treatment unit for a 38-villa community (~ 152 residents) in Dubai, coupling the two systems exclusively through the electricity demand of the treatment plant rather than conducting a dual-resource economic assessment. Gu et al.^19^ modelled PV together with wastewater recycling at the individual smart-home level as an energy scheduling optimization problem, a fundamentally different framework from the infrastructure-scale assessment conducted here. Abdelhamid et al.^20^ coupled solar energy with treated greywater for hydroponic production, addressing the water–energy–food nexus rather than urban residential resource efficiency. Odeh R ^21^. established that greywater reuse for toilet flushing and irrigation can significantly reduce freshwater demand in water-stressed regions, while Batisha^9^ quantified the greywater reuse potential in Egypt at 4.15–8.30 billion m^3^/year, underscoring the national-scale relevance of compound-level implementations such as the one examined here. Collectively, these studies confirm the technical viability of greywater reuse and PV deployment in residential settings but do not provide a compound-scale, dual-resource assessment with disaggregated capital and operating costs benchmarked against published MENA data—the specific gap this study addresses.

Expanding these findings, this study proposes a novel solution by evaluating an integrated resource-efficiency concept for a residential compound in New Cairo, Egypt, combining treated greywater reuse for non-potable demands (with toilet flushing as the primary quantified end use) and rooftop solar PV to reduce reliance on potable water supply and grid electricity. The analysis is developed using the project inventory and stated per-capita end-use assumptions to derive baseline water flows and the corresponding offset potential.

The present work contributes a reproducible, project‑inventory–based assessment at residential compound scale in New Cairo, Egypt. Using the development inventory (365 buildings; 7,512 units; ~ 45,000 cap) and stated per‑capita end‑use values, the study:

- Quantifies a demand-limited potable-water offset for toilet flushing derived from explicit baseline flows.
- Reports replicable design and operational parameters for a 6,300 m^3^ day^−1^ greywater treatment system.
- Presents a disaggregated CAPEX split (in-building supply, networks, treatment plant) to support verification and comparability.
- Conducts a unified ± 20% sensitivity analysis covering water (occupancy, reuse fraction) and PV (yield factor) within one internally consistent dataset.

Therefore, the novelty of this approach lies in four specific and verifiable contributions: (1) a compound-scale integrated assessment (365 buildings; ~ 45,000 residents) that is substantially larger than comparable published greywater–PV studies; (2) a fully reproducible, step-by-step methodology anchored to the Egyptian Code and stated per-capita end-use values, enabling independent verification; (3) a disaggregated CAPEX and OPEX structure benchmarked against, supporting cross-study comparability; and (4) a unified ± 20% sensitivity analysis covering occupancy, reuse fraction, and PV yield within one internally consistent dataset, consistent with published approaches for decentralized greywater cost–benefit assessment.

## Project description and methodology

### Study area

The study area, Jana residential compound in New Cairo, comprises a total of 365 buildings, each consisting of a ground floor and five repeated floors. In both Model A and Model B configurations, each floor contains four apartments. Model A apartments include two bathrooms and a kitchen, while Model B apartments feature three bathrooms and a kitchen.

The buildings are categorized into two main types:

- 287 buildings with a total area of 490 m^2^, each containing two apartments of 115 m^2^ and 22 apartments of 130 m^2^.
- 78 buildings with a total area of 330 m^2^, each containing one apartment of 100 m^2^, eleven apartments of 130 m^2^, ten apartments of 140 m^2^, and two apartments of 150 m^2^.

The compound extends over approximately 200 feddans, with around 20 feddans allocated for landscaping, including green spaces and parks.

The buildings are categorized as summarized in Table 1.

**Table 1:** Residential Building Configurations.

Model type	Unit area (m^2^)	Units per building	Total units per model
Model A	115	2	574
		22	6,314
Model B	100	1	78
	130	11	234
	140	10	156
	150	2	156
Total	–	–	7,512

The project spans a total area of approximately 200 feddans, as detailed in Table 2.

**Table 2:** Land use distribution.

Item	Area (m^2^)	Percentage (%)
Ground floor footprint (FP)	27,633	15.1
Roads and parking	53,070	29
Green areas and parks	89,670	49
Utilities and pedestrian paths	12,627	6.9
Total project area	183,000	100

### Greywater reuse system

Greywater is challenging to classify as a specific wastewater type due to its variable composition and source. Generally, its organic strength is comparable to that of low- to medium-strength municipal sewage and shares characteristics with tertiary-treated effluent in terms of biodegradability and physical pollutants^18^. The primary challenge in treatment lies in its organic content, which impacts the appearance, safety, and regulatory compliance of the reused water. A particular health concern is the chlorine demand caused by residual organics, which can compromise disinfection effectiveness if not properly managed^19^.

Greywater refers to wastewater from domestic sources like kitchens, bathrooms, and laundry, excluding toilet discharge, which is classified as blackwater^20,21^. It contains substances such as soap, shampoo, toothpaste, food scraps, and oils. Based on pollution levels, greywater can be categorized as low, moderate, high, or mixed—where mixed greywater is generally the most polluted due to source combination^22^. Understanding its characteristics is essential for selecting appropriate treatment technologies and assessing potential health and environmental risks^3^.

The volume of greywater produced varies depending on the socio-economic conditions of each country. For instance, low-income countries may produce as little as 20–30 L per person per day, whereas in most cases, the typical range lies between 80 and 120 L per person per day^18,21,22^. In terms of quality, greywater contains various pollutants including organic carbon—measured as BOD, COD, and TOC—suspended solids, nutrients such as nitrogen and phosphorus, surfactants, as well as emerging contaminants from personal care products and certain pharmaceuticals^13,23^. Greywater generally makes up the largest portion of household wastewater, contributing approximately 50–80% of the total domestic flow^21^. The composition varies with its origin; for example, water from showers, bathtubs, and hand basins tends to be less polluted compared to that from kitchen sinks or laundry machines^24^. Due to its warm temperature and the presence of nutrients, greywater can quickly degrade in quality when stored, creating favorable conditions for pathogen growth. Therefore, timely and appropriate treatment is essential to reduce associated health risks and enable safe reuse^25^.

**Fig. 1:**
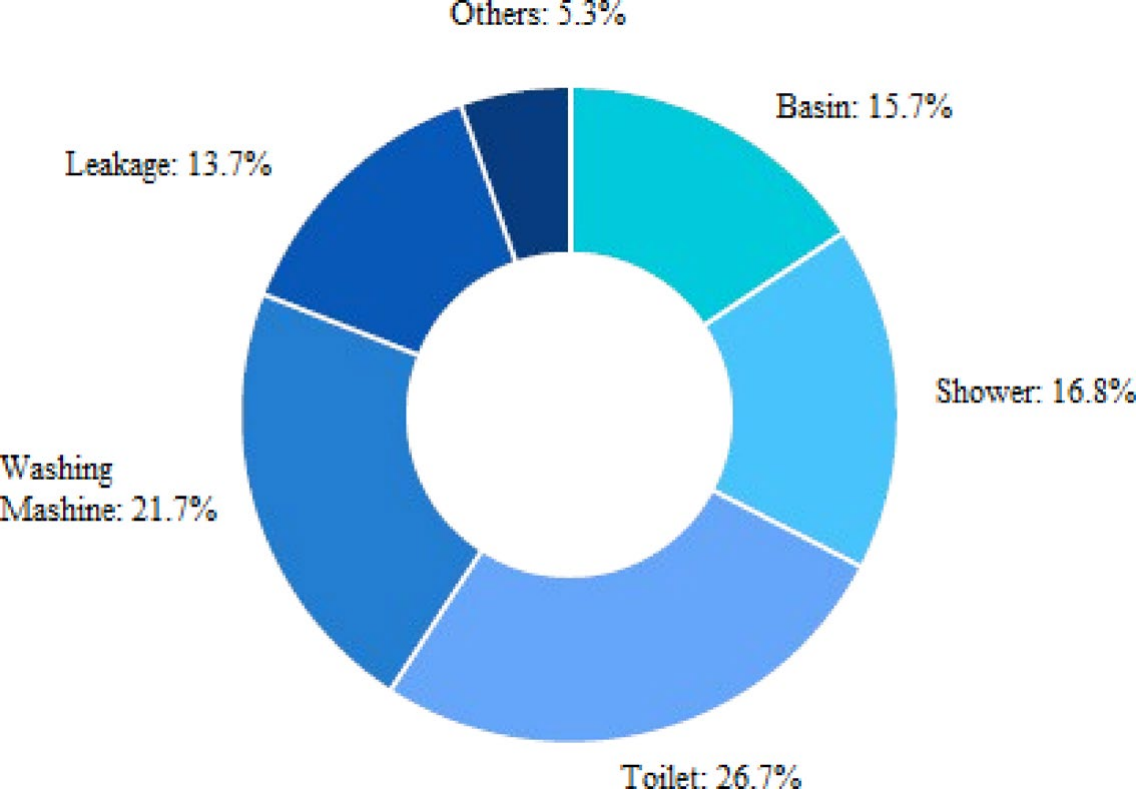
Indoor per capita water use percentage including leakage.

**Fig. 2:**
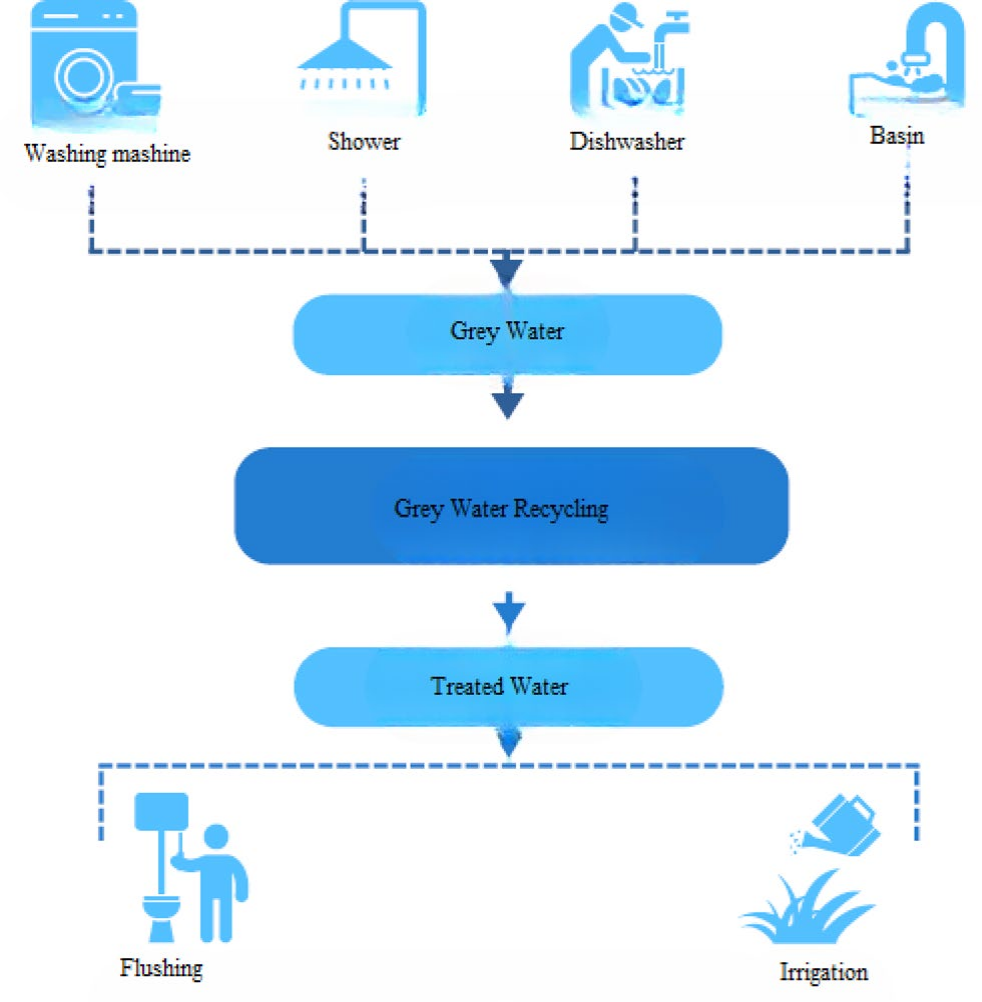
Greywater recycling process.

**Fig. 3:**
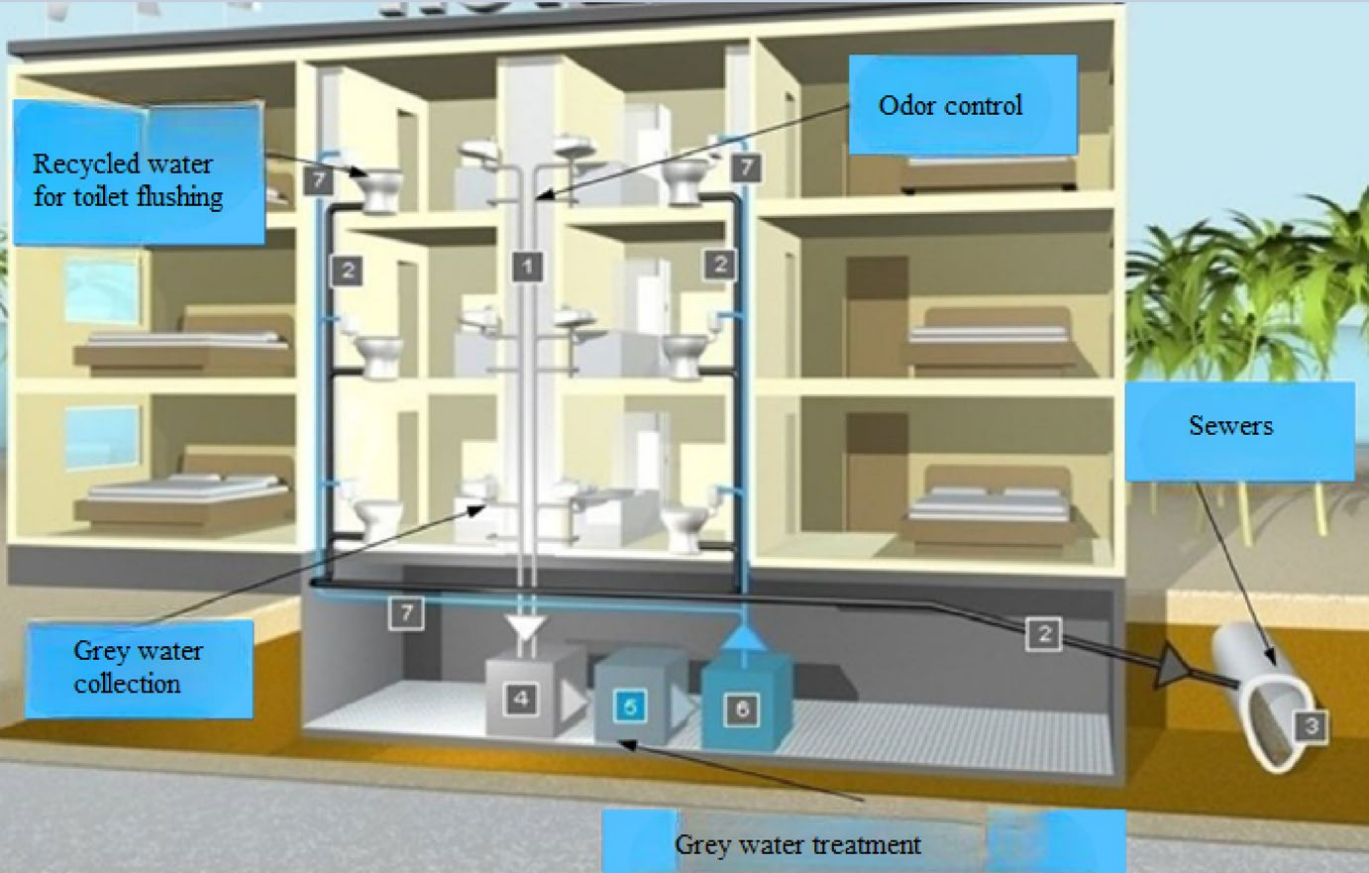
greywater cycle within the building.

Figure 1 illustrates the Indoor per capita water use percentage including leakage. According to the American Water Works Association Research Foundation^26^, indoor water use was distributed among various fixtures: toilets accounted for 26.7%, clothes washers 21.7%, showers 16.8%, faucets (basins) 15.7%, leaks 13.7%, and other sources 5.3%. While the figure includes all indoor water uses, for greywater-focused analysis, only contributions from clothes washers, showers, basins, and similar non-toilet sources are considered relevant. Greywater constitutes the largest portion of household wastewater. However, if discharged untreated, it may reduce oxygen levels in water bodies and stimulate microbial activity, leading to water quality degradation. Despite these risks, properly managed greywater can be reused effectively in non-potable applications. Recent years have seen increased international interest in greywater reuse, with growing recognition of its potential to relieve pressure on freshwater esources^24,25,27^. After appropriate treatment (Fig. 2), greywater can be safely reused. Figure 3 illustrates the greywater cycle within the building.

#### Greywater and black water characteristics

**Fig. 4:**
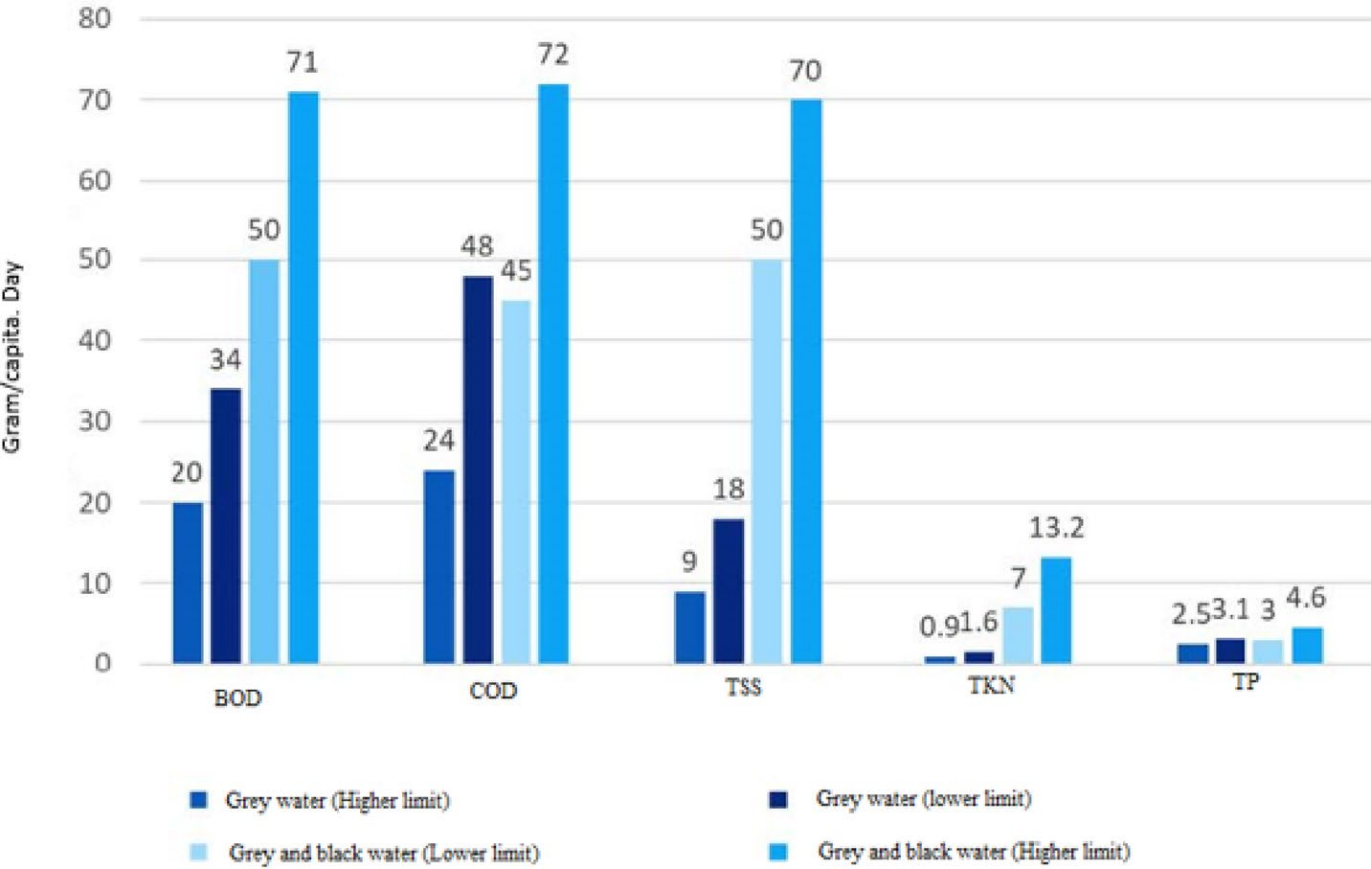
Characteristics of greywater and black water.

Greywater from domestic sources contains fewer pollutants than blackwater and is generally easier to treat. The comparative model is based on pollutant load differentials and treatment complexity indicators while, assessment of treatment difficulty based on organic load, nutrient concentration and pathogen density. Laboratory experiments focused on physicochemical and biological characterization of greywater& blackwater and the evaluation of treatment efficiency. Parameters analyzed included pH, electrical conductivity, turbidity, total suspended solids, biochemical oxygen demand, chemical oxygen demand and indicator microorganism. Figure 4 presents a comparison of their characteristics by source and contaminant level.

### Solar PV modeling and representation

Solar PV system performance is primarily influenced by ambient temperature (T_a_) and sun irradiance (G), which are the main determinants of its output power (P_PV_). This output can be calculated using Eq. (1) ^28^:

1 $${P}_{PV}={P}_{PV, STC}\cdot \frac{G}{{G}_{STC}}\cdot \left(1+{K}_{t}\left({T}_{a}+0.0256\cdot G-{T}_{STC}\right)\right)\cdot{\eta }_{MPPT}$$

*T*_*a*_: Ambient Temperature ($^\circ C$), *G*: Solar irradiance ($W/{m}^{2}$), ${P}_{PV}$ : The output power of the solar PV at a certain operating Temperature (*T*_*a*_), and solar irradiance (*G*), ${G}_{STC} and {T}_{STC}$ : the standard test condition (STC) of solar irradiance ($1000 W/{m}^{2}$) and temperature ($25^\circ C$). ${P}_{PV, STC}$: the solar rated output power under STC, ${K}_{t}$ : the temperature coefficient, ${\eta }_{MPPT}$ : the efficiency of maximum power point tracking point (assumed to be 98% in this study). Assuming the ambient temperature stays at 25 °C, the PV power merely changes in a linear fashion with G. For the irradiance dataset, it has been selected by PVsyst which is a software tool designed for the solar energy industry. The database library of PVsyst has different irradiance profile based on the geographical location that has been selected based on the study area. PVsyst has different brand models for PV solar system that reflect real manufactures, Suntech Power has been selected as it is long-standing manufacturer of crystalline silicon modules.

Suntech Power modules, particularly the Ultra V Pro N-type (545-570W) and670W series, are designed for high-efficiency, large-scale residential, commercial, and utility projects. Based on typical installation guidelines and industry standards, the following are the recommended DC/AC sizing values and design parameters for systems utilizing Suntech panels: Industry standards recommend a DC/AC ratio (the ratio of total solar panel DC capacity to inverter AC capacity) greater than 1.0 to maximize inverter utilization, with ideal ratios for Suntech systems typically falling between 1.1 and 1.5. Suntech Power has an annual Degradation for newer Ultra V Pro panels, where the warranty promises less than 1% degradation in the first year and 0.4% annually in subsequent years. About the Long-Term Performance, after 30 years, Ultra V Pro panels are warranted to maintain at least 87.4% to 89.2% of their original capacity. For power tolerance, Suntech modules typically operate within a -0/+5 W range or ±3% of the rated power (Pmax) under Standard Test Conditions (STC). Where mismatch losses, its current sorting techniques in manufacturing reduce power loss caused by current mismatch to roughly 2%.

**Fig. 5:**
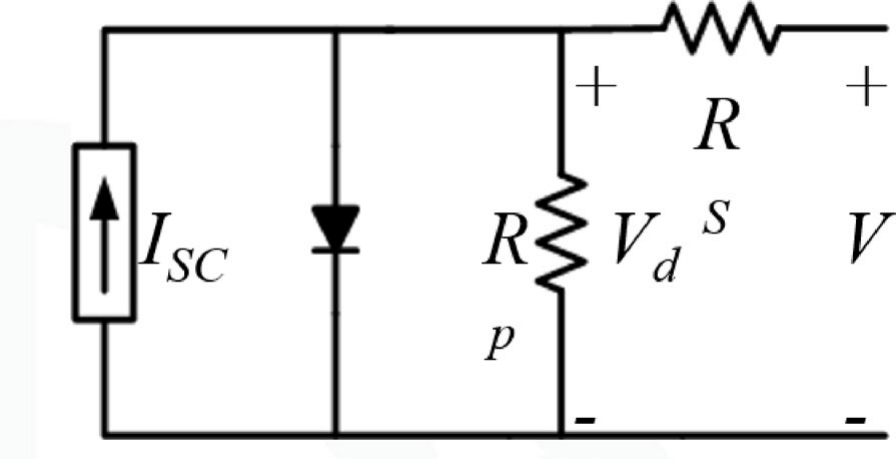
Equivalent modeling of solar PV system.

As a result, and for a simpler representation, the PV system may also be represented using a first-order transfer function, as shown in Eq. (2) to link between the outcome of output power and the input of solar irradiance based on the change rate (Δ) of both of them^29^:

2 $$T.{F}_{PV}=\frac{\Delta {P}_{PV}}{\Delta G}=\frac{{K}_{PV}}{1+s\cdot{\tau }_{PV}}$$

where $T.{F}_{PV}$: transfer function of the photovoltaic (PV) system, $\Delta {P}_{PV}$: incremental change in PV output power, $\Delta G$: incremental change in solar irradiance, ${K}_{PV}$: static gain of the PV system, S: Laplace operator and ${\tau }_{PV}$: time constant of the PV system, where ${K}_{PV} and {\tau }_{PV}$ are PV system gain and time constant, respectively. This behavior can be represented in an equivalent circuit model, as illustrated in Fig. 5.

### Environmental and strategic dimensions of the system

Egypt is currently facing mounting challenges in ensuring a reliable supply of freshwater. These pressures are largely driven by rapid urban development and the uncertain impacts of the Grand Ethiopian Renaissance Dam on Nile River flows. Global projections add to this urgency: UNICEF^30^ reports that by 2040, nearly 600 million children are expected to live in areas with extremely limited water resources, while FAO^31^ estimates that 1.8 billion people may endure absolute water scarcity, with two-thirds living under water-stressed conditions. These realities highlight the need for urgent action to expand water resources and safeguard existing supplies. Among the most promising strategies is the reuse of domestic wastewater through modern treatment technologies—an approach that is increasingly viewed not just as a technical option, but as a national necessity.

In response, the Egyptian government and its key institutions have launched several projects to protect and better manage freshwater resources. These efforts emphasize the adoption of advanced wastewater treatment systems. Globally, greywater reuse is gaining momentum as a practical solution to ease pressure on freshwater systems, especially in regions facing similar challenges. As part of broader goals to conserve energy and use resources more efficiently, many Egyptian institutions are now adopting greywater reuse practices. These initiatives are seen as beneficial not only for reducing consumption but also for delivering meaningful environmental, economic, and social gains. Under the current national conditions, such systems are playing an increasingly strategic role in supporting Egypt’s sustainable development.

Notably, several greywater reuses projects—both experimental and operational—have already been implemented in different parts of the country. Their growing presence reflects an expanding commitment to integrating greywater systems into Egypt’s future water management plans.

## Technical study

### Derivation of greywater flow rates

The expected greywater production for the Janna compound is derived through a transparent, step by step methodology based on the Egyptian Code and established international benchmarks.

#### Population determination

Based on Table 1, the total number of units is 7,512. Assuming an average of six residents per unit, the total population is approximately 45,000 capita.

#### Hydrological assumptions and per capita use

The derivation relies on the following assumptions for residential water and wastewater cycles:

- *Potable Consumption*: The daily water consumption per capita is approximately 250 L/day, according to the Egyptian Code for luxury residential categories.
- *Wastewater Generation*: The average flow of wastewater is around 200 L/c/d (representing an 80% return-to-sewer factor^26^).
- *Greywater Fraction*: Greywater (excluding toilet discharge) typically constitutes between 60 and 80% of the total household wastewater flow.
- *Greywater Recovery Potential*: The recovery rate ranges from 120 to 160 L/c/d, with an average of about 140 L/c/d available for reuse.
- *Toilet Flushing Demand*: The average daily consumption for flushing toilets is approximately 60 L/c/d per person (representing 30% of the total sewage flow).

#### Calculation of daily volumetric totals

Using the parameters defined above, the total daily flows for a population of 45,000 are calculated as follows:

- $$\begin{aligned} {\mathrm{Total}}\;{\mathrm{potable}}\;{\mathrm{water}}\;{\mathrm{demand}}\left( {Q_{{total}} } \right) & = 45,000\;{\mathrm{cap}} \times 250\;{\mathrm{L}}/{\mathrm{c}}/{\mathrm{d}} \\ & = 11,250,000\;{\mathrm{L}}/{\mathrm{d}} \\ & = 11,250\;{\mathrm{m}}^{3} /{\mathrm{d}} \\ \end{aligned}$$  
- $$\begin{aligned} {\mathrm{Total}}\;{\mathrm{wastewater}}\;{\mathrm{flow}}\left( {Q_{{ww}} } \right) & = 45,000\;{\mathrm{cap}} \times 200\;{\mathrm{L}}/{\mathrm{c}}/{\mathrm{d}} \\ & = 9,000,000\;{\mathrm{L}}/{\mathrm{d}} \\ & = 9,000\;{\mathrm{m}}^{3} /{\mathrm{d}} \\ \end{aligned}$$  
- $$\begin{aligned} {\mathrm{Total}}\;{\mathrm{toilet}}\;{\mathrm{flushing}}\;{\mathrm{demand}}\left( {Q_{{{\mathrm{flushing}}}} } \right) & = 45,000\;{\mathrm{cap}} \times 0.06\;{\mathrm{m}}^{3} /{\mathrm{c}}/{\mathrm{d}} \\ & = 2,700\;{\mathrm{m}}^{3} /{\mathrm{d}} \\ \end{aligned}$$  
- $$\begin{aligned} {\mathrm{Estimated}}\;{\mathrm{greywater}}\;{\mathrm{production}}\left( {Q_{{{\mathrm{gw}}}} } \right) & = 45,000\;{\mathrm{cap}} \times 140\;{\mathrm{L}}/{\mathrm{c}}/{\mathrm{d}} \\ & = 6,300,000\;{\mathrm{L}}/{\mathrm{d}} \\ & = 6,300\;{\mathrm{m}}^{3} /{\mathrm{d}} \\ \end{aligned}$$  

Based on this derivation, the estimated greywater production is 6,300 m^3^/day. Therefore, the required capacity of the treatment plant is also 6,300 m^3^/day.

### Greywater treatment

The greywater treatment section outlines strategies intended to improve water quality and ensure safe reuse in potential future implementation. The proposed treatment involves filtration, biological processes, and disinfection steps that can be integrated within residential buildings. These processes support a range of reuse options, and the main goals of the treatment system are:

- Ensuring water quality aligns with intended uses.
- Meeting public health standards for treated greywater.
- Conserving freshwater resources by reducing overall consumption.
- Lowering the overall water-related operational costs

The table below outlines the properties of treated greywater and its suitability for reuse (Table 3).

**Table 3:** Properties of Treated Greywater and its Suitability for Reuse [(adapted from NSF International & ANSI, 2023, NSF/ANSI 350:2023 ^32^: Onsite Residential and Commercial Water Reuse Treatment Systems, Ann Arbor, MI: NSF International)].

Property	Treatment Quality	
	Test average	Single sample maximum
Turbidity	5	10
pH	6–9	NA
BOD_5_ (mg/L)	10	25
TSS (mg/L)	10	30
Residual Chlorine (mg/L)	0.5‒2.5	NA
E. coli (MPN/100 mL)	14	240

The table ensures treated greywater meets specific quality levels and highlights its potential applications based on health and environmental standards.

#### Process flow schematic

**Fig. 6:**
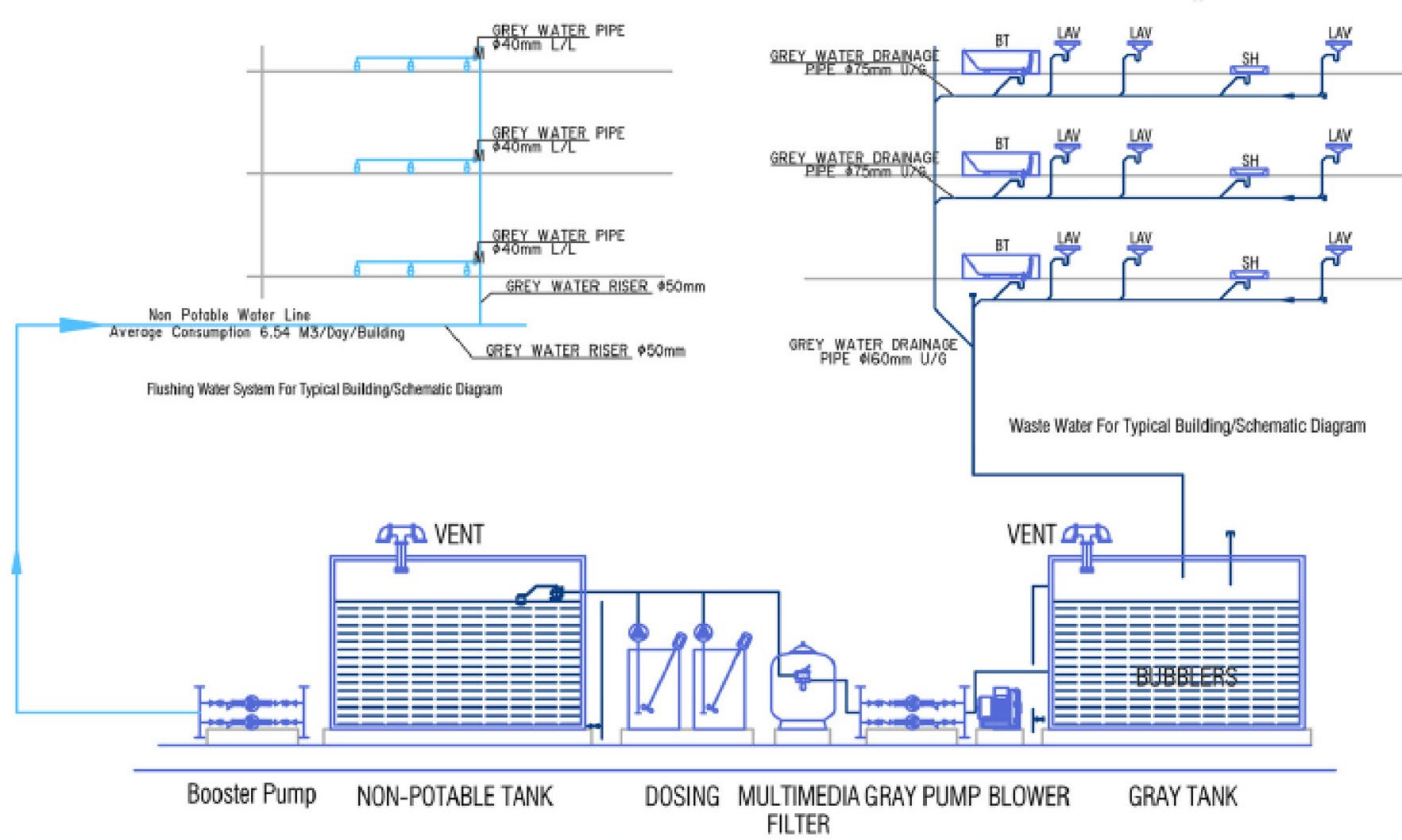
Detailed schematic of greywater system components.

The greywater treatment system involves multiple stages, with treatment quality depending on the specific reuse application. As illustrated in Fig. 6, the process flow begins with collection in a dedicated tank, followed by dosing coagulant, multi-media filtration, and chlorine dosing before the reclaimed water is stored in the final tank for distribution.

#### Technical design and operational parameters

The 6,300 m^3^/day facility is designed based on the following parameters to ensure reproducibility and system reliability (Table 4).

**Table 4:** Technical design and operational specifications:

Component/Process	Parameter	Design value	Rationale/Standard
Non-potable tank	Total Volume (m^3^)	2,100	8-h HRT for flow equalization
Multimedia Filtration	Filtration Rate (m^3^/m^2^/d)	300	180–400 m^3^/m^2^/d according to Egyptian Code
Disinfection Unit	Chlorine Dosage (mg/L)	5.0– 20.0	according to Egyptian Code
Grey Tank (Treated Water Storage)	Storage Duration	12 Hours	Buffer for toilet flush cycles

#### Quality monitoring plan

To ensure safe and consistent operation of the treated greywater system, a monitoring program is implemented combining routine field measurements and periodic laboratory analyses. Routine monitoring focuses on parameters that provide immediate indication of treatment stability and disinfection effectiveness (turbidity, pH, and residual chlorine), while laboratory testing confirms organic removal performance and microbiological safety (BOD_5_/TSS and indicator organisms) (Tables 5, 6).

**Table 5:** Minimum treated greywater monitoring and corrective action plan:

Parameter	Location	Frequency	Target/action limit	Corrective action
Turbidity (NTU)	Filter effluent / storage inlet	Daily (field/online)	Maintain within reuse targets (e.g., < 5 NTU)	Check filter integrity/backwash; divert off-spec water to sewer
pH	Storage tank	Daily	6–9	Adjust dosing; inspect chemical feed
Free/total residual chlorine (mg L⁻^1^)	Storage tank + user end	Daily	Maintain residual in storage at 0.5‒2.5 mg/L	Adjust chlorine dose
BOD_5_ (mg L⁻^1^)	Treated water	Weekly (lab)	(BOD_5_ < 10 mg L⁻^1^)	Investigate upstream loading
TSS (mg L⁻^1^)	Treated water	Weekly (lab)	(TSS < 10 mg L⁻^1^)	Investigate upstream loading

**Table 6:** Monthly greywater generation and recovery (Modeled Results).

Month	Days	Design flow (m^3^/day)	Monthly generation (m^3^)	Recovery efficiency (%)	Monthly recovery (m^3^)
January	31	6,300	195,300	95.0	185,535
February	28	6,300	176,400	95.0	167,580
March	31	6,300	195,300	95.0	185,535
April	30	6,300	189,000	95.0	179,550
May	31	6,300	195,300	95.0	185,535
June	30	6,300	189,000	95.0	179,550
July	31	6,300	195,300	95.0	185,535
August	31	6,300	195,300	95.0	185,535
September	30	6,300	189,000	95.0	179,550
October	31	6,300	195,300	95.0	185,535
November	30	6,300	189,000	95.0	179,550
December	31	6,300	195,300	95.0	185,535
ANNUAL TOTAL	365	6,300	2,299,500	95.0	2,184,525

### System components

The provision of the building’s internal sanitary system includes additional works in the case of using a greywater system, as follows:

- *Pipes*: Greywater supply pipes to feed toilet flushing tanks, including all necessary fittings and connections.
- *Networks*: Collection and transfer of greywater from its sources to the greywater treatment plant.
- *Lines*: Lines for transferring treated greywater that meets the required specifications to designated usage sites, such as irrigation networks within the compound or for watering green landscaping areas.
- *Storage Tanks*: Tanks to store treated greywater to ensure an adequate supply.
- *Analysis and Monitoring Units*: Monitoring the treated greywater to ensure compliance with reuse standards^33^.
- *Pumping Systems*: Transport treated water to its point of use.

The following alternatives are considered in designing the system for greywater reuse within residential buildings:

#### First alternative

This option involves installing a conventional greywater system in residential complexes. It outlines system components and their interconnections, shown in Fig. 6. Greywater from household fixtures is collected and treated separately for reuse in irrigation and possibly other non-potable applications.

**Fig. 7:**
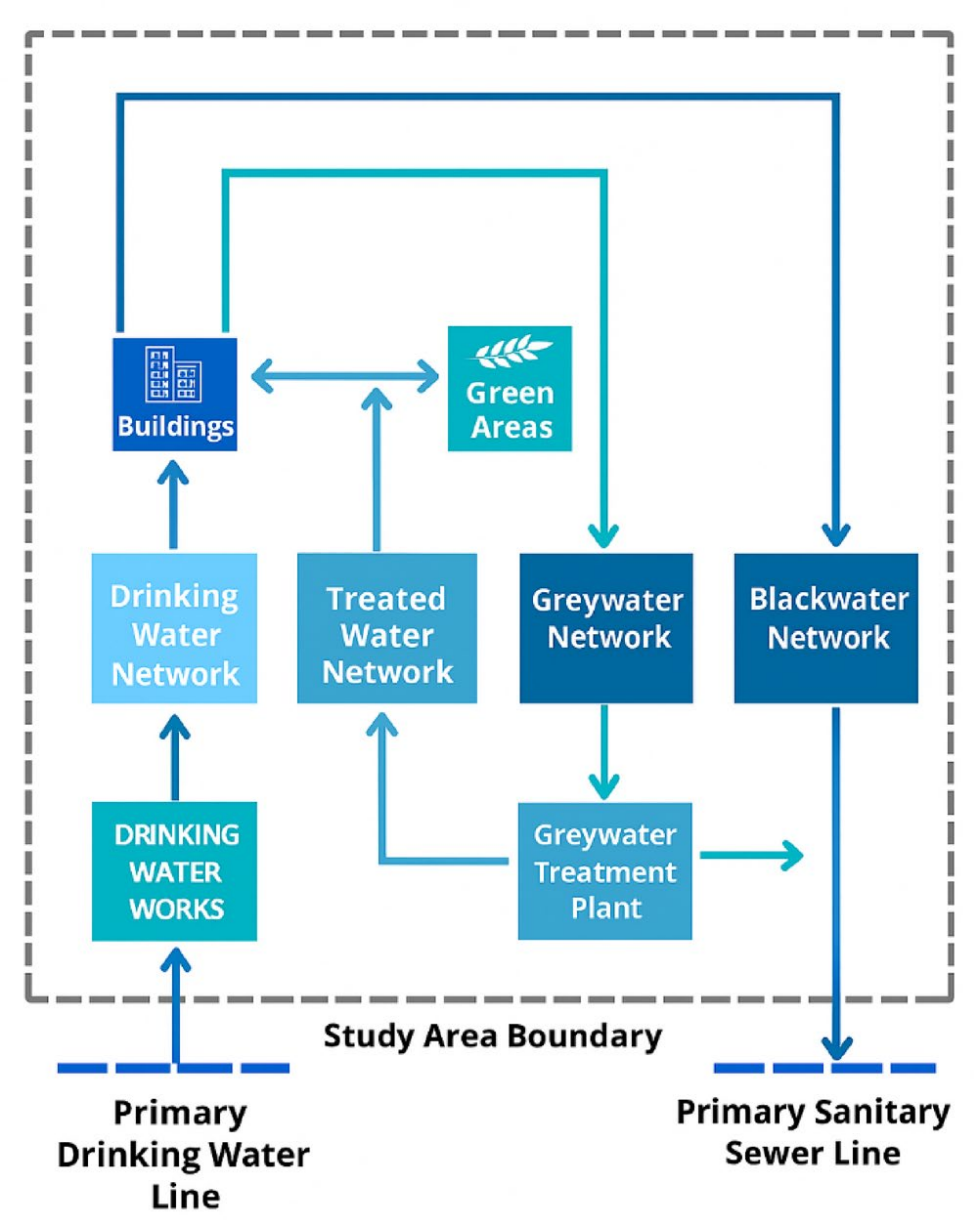
Water cycle in the project with traditional alternative.

Figure 7 shows how greywater is treated and reused within the project for internal applications and irrigation.

#### Second alternative

Given the existence of a tertiary-treated wastewater reuse system that meets the technical specifications and global quality standards for reuse in various residential communities, and considering the availability of infrastructure and treated water supply lines reaching the project area, the following approach is proposed:

- Establishing a network for transporting treated wastewater that meets the required specifications and ensures adequate pressure levels for distribution to buildings. This network will enable the reuse of treated water in toilet flushing systems across different buildings within the development.
- Constructing networks for greywater to a dedicated greywater treatment plant within the project.
- Developing a greywater treatment plant equipped with storage tanks and pumping systems to facilitate the reuse of treated greywater for irrigating green areas.
- Connecting treated greywater distribution pipelines to irrigation tanks inside greywater treatment plants to fulfill the central requirements of irrigation systems.

**Fig. 8:**
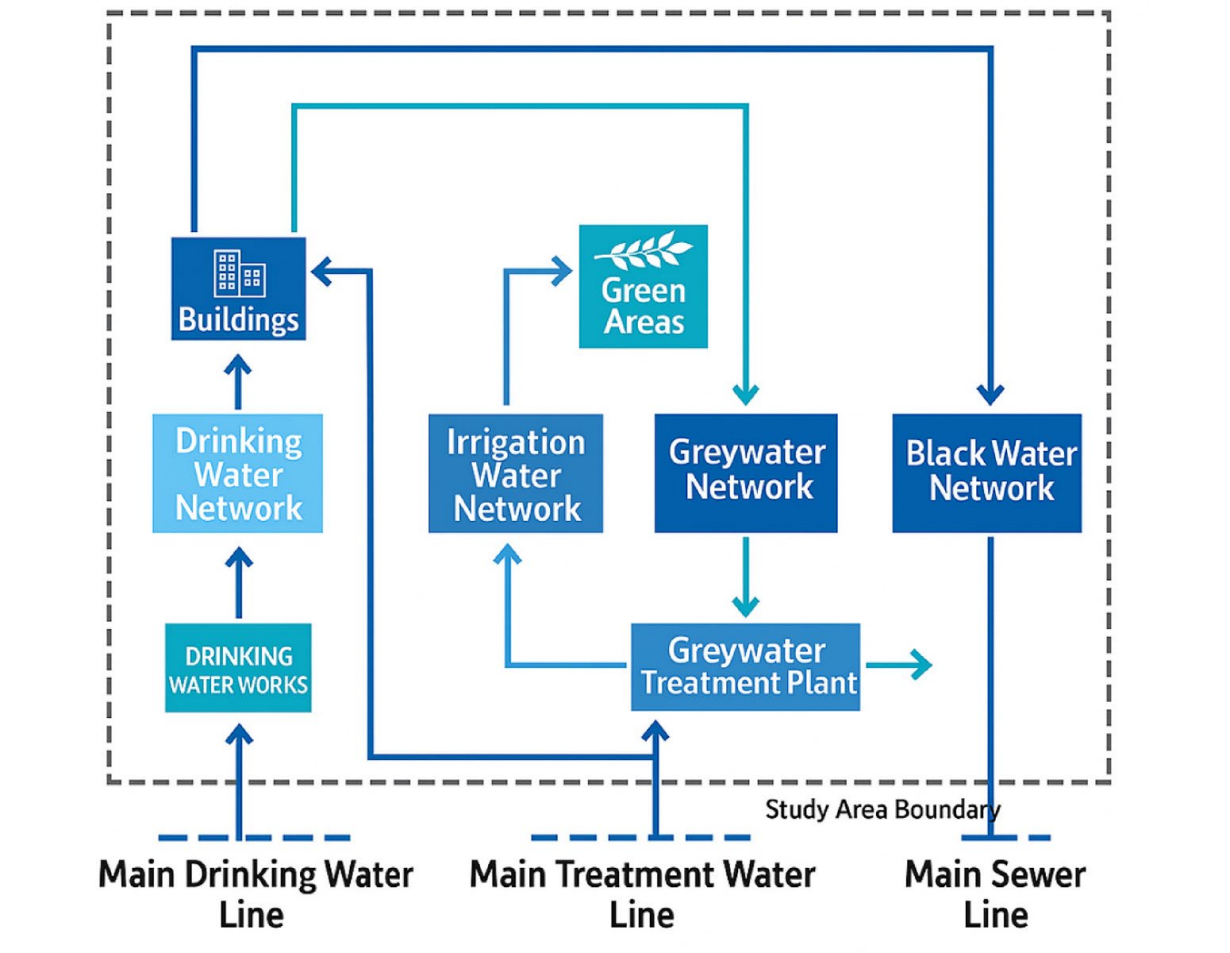
Water cycle in the second alternative.

Figure 8 illustrates the water cycle within the project under the second alternative, where a portion of wastewater (greywater) is treated and reused in household applications and irrigation purposes:

### Modeled (design-stage) monthly and annual profiles

Treatment Technology: The greywater treatment system employs a physio‑chemical treatment train consisting of multimedia filtration, coagulation/flocculation, and chlorination. Such treatment trains have been widely reported for onsite greywater reuse applications because they provide robust removal of suspended solids and turbidity with relatively simple operation compared with purely biological systems^12,13^. In this design‑stage model, backwash water was assumed as 5% of influent flow, consistent with reported typical values around ~5% and published ranges of approximately 2–10% depending on plant practice^34^.

#### Monthly greywater generation and recovery profiles

Table 7 presents the modeled monthly greywater generation and recovery profiles for the Jana residential compound. The system is modeled at a constant influent flow of 6,300 m^3^/day, and recovery is calculated from the assumed backwash fraction (i.e., hydraulic recovery = 1 − backwash fraction).

**Table 7:** Estimated costs of the greywater system.

Component	Specific CAPEX (USD/(m^3^/day))	CAPEX (USD)
Greywater Supply System for Feeding Toilet Flush Tanks in Residential Buildings	105	670,000
Greywater collection and transfer networks to and from the treatment plant	160	1,000,000
Greywater Treatment Plant	270	1,700,000
Total Cost for Greywater System	535	3,370,000

#### Key findings

- Total annual greywater generation: 2,299,500 m^3^/year, equivalent to 6,300 m^3^/day based on a population of 45,000 residents at 140 L/capita/day.
- Total annual greywater recovery: 2,184,525 m^3^/year, representing 95% of generated greywater.
- Backwash water consumption: 114,975 m^3^/year (5% of influent), used for periodic filter cleaning.
- The recovered greywater (≈2.18 million m^3^/year) exceeds the annual toilet flushing demand computed from the project baseline assumptions (2,700 m^3^/day × 365 days = 985,500 m^3^/year), indicating surplus treated volume under the modeled conditions; the allocation of any surplus to other end uses would require separate demand definition and quality compliance assessment.

## Financial study

The amount of greywater requiring treatment corresponds to the estimated daily generation of approximately 6,300 m^3^. To improve transparency and reproducibility, the financial assessment is presented in terms of (i) capital expenditure (CAPEX) expressed as total cost and as specific cost per unit design capacity (USD/(m^3^/day)), and (ii) operating expenditure (OPEX) expressed as unit cost per cubic meter of greywater (USD/m^3^) and the corresponding annual cost at the design flow.

Total costs are separated into (a) greywater supply within buildings, (b) collection and transfer networks to and from the treatment facility, and (c) the greywater treatment plant. OPEX is disaggregated into energy, labor, chemicals/disinfectants, maintenance and repairs, and materials/consumables and quality monitoring to support reproducibility and comparison across studies.

The construction cost for treating one cubic meter of greywater ranges between 160 and 300 USD. For operating expenditure (OPEX), evidence from facilities in the Middle East and North Africa (MENA) reports operational costs for tertiary treatment plants of 0.03–0.09 USD/m^3^ across several facilities, and 0.022 USD/m^3^ for a large tertiary activated‑sludge facility in Egypt^35^. As an additional benchmark outside MENA, a detailed cost assessment for tertiary treatment plus disinfection in the European Mediterranean context reported total operating costs of 0.069–0.154 EUR/m^3^, depending on plant scale and cost scenario^16^. In this study, OPEX is itemized into (i) routine operation and compliance activities (operator time, routine inspections, and baseline water‑quality monitoring) and (ii) utilities/consumables and minor maintenance. The routine operation and compliance cost is set at 0.03 USD/m^3^. Additional allowances of 0.02 USD/m^3^ for energy and 0.01 USD/m^3^ for other consumables/maintenance are included (Table 8), which correspond to the energy and “other” cost magnitudes reported for tertiary reuse plants in MENA.

**Table 8:** Operating expenditure (OPEX) for greywater treatment.

OPEX item	Reference	Unit cost (USD/m^3^)	Annual cost (USD/year)
Routine operation & compliance (operator time, routine inspections, baseline QA/QC)	Adopted in this study; benchmarked against reported OPEX for tertiary reuse plants in Egypt/Tunisia.^35^	0.03	68,985
Energy (electricity for treatment and transfer pumping)	Energy cost component reported as 0.02 USD/m^3^ for South Sfax tertiary plant (Table 4.3) ^35^	0.02	45,990
Other consumables & minor maintenance (e.g., small spares, routine materials)	‘Others’ cost component reported as 0.01 USD/m^3^ for tertiary plants (Table 4.3) ^16^	0.01	22,995
Total OPEX	Sum of above items; consistent with published tertiary-treatment + disinfection OPEX magnitudes[36; 37]	0.06	137,970

Table 7 summarizes the CAPEX of the greywater system by major components, and Table 8 reports the OPEX in unit and annual terms. Each unit OPEX item in Table 8 is linked to published cost components for tertiary treatment/reuse facilities in MENA^35^.

### Levelized cost of water (LCOW)

LCOW is defined as the annualized cost of the greywater system divided by an annual water volume^36^. A transparent straight‑line annualization over the 20‑year analysis horizon used in the return assessment is reported here:

$$L{\mathrm{COW}}_{{{\mathrm{SL}}}} \, = \,\left( {{\mathrm{CAPEX}}/L\, + \,{\mathrm{OPEX}}_{{{\mathrm{annual}}}} } \right)/V_{{{\mathrm{annual}}}}$$

**Table:** 

Metric	Value	Unit
CAPEX (greywater system total)	3,370,000	USD
Total OPEX (unit)	0.0600	USD/m^3^
Total OPEX (annual)	137,970	USD/year
Annual treated volume	2,299,500	m^3^/year
Annual potable offset (toilet flushing only)	985,500	m^3^/year
LCOW_SL (treated‑volume basis)	0.133	USD/m^3^
LCOW_SL (offset basis; flushing only)	0.311	USD/m^3^

Because toilet flushing is demand‑limited in the baseline case, LCOW expressed per m^3^ of potable offset is higher than LCOW per m^3^ treated; allocating treated greywater to additional non‑potable end uses would increase potable offset and reduce LCOW_offset_.

### Solar energy

Solar energy stands as a prominent alternative and renewable energy source, distinguished by its unparalleled abundance among all forms of renewable energy. It retains its potential even under overcast weather conditions.

Solar energy technologies serve a wide range of applications, including heating, cooling, lighting, and electricity generation, all derived from natural sunlight. These technologies convert sunlight into either electrical energy—typically via photovoltaic cells—or thermal energy using mirrors that concentrate solar radiation. Egypt has very high solar radiation levels, among the highest in the world, due to its geographic location (most desert and arid climates), long sunshine hours, and relatively clear skies. This makes it highly suitable for a wide range of solar technologies. Typical sunshine duration ranges from (6–11) hours/day in north regions and (9–12) hours/day in southern and desert regions.

The cost of solar panels has decreased significantly over the past decade, making solar energy one of the most affordable renewable energy options. On average, solar panels remain in service for around 30 years, although their efficiency depends on the materials used in their manufacture.

According to the International Renewable Energy Agency (IRENA), between 2010 and 2023, the weighted-average levelized cost of electricity decreased by 70% for concentrated solar power (CSP), 90% for solar photovoltaic (PV), 63% for offshore wind farms, 70% for onshore wind farms, and 14% for bioenergy. Conversely, it increased by 33% for hydropower and 31% for geothermal energy. The IRENA report on global renewable energy expansion over the last decade is summarized in Table 9^37,38^.

**Table 9:** The global renewable expansion over the last decade.

RESs	Year 2010 generation (GW)	Year 2010 Weighted-average cost of electricity (USD/kWh)	Year 2023 generation (GW)	Year 2023 Weighted-average cost of electricity (USD/kWh)
Solar PV	42	0.460	1411	0.044
CSP	> 1	0.393	6.9	0.117
Offshore Wind	3.1	0.203	73.2	0.075
Geothermal	10	0.054	15	0.071

#### Advantages of using solar energy

The adoption of solar energy provides several advantages that support the global transition toward clean and sustainable energy sources:

- Environmental benefits of solar energy:

- *Renewable and Sustainable Source*: Solar energy is considered one of the renewable and sustainable energy sources. It depends on an inexhaustible source of energy, which helps reduce dependence on expensive and limited conventional energy sources over the long term. Solar energy is also considered a clean and non-polluting source of energy.
- *Helps Reduce Global Warming*: Solar energy reduces carbon emissions since it does not produce any emissions or air pollutants.
- *Reduction of Greenhouse Gas Emissions*: It does not generate harmful gases such as carbon dioxide, making it a key solution for reducing the carbon footprint.
- *Reduces Dependence on Non-Renewable Resources*: It reduces the depletion of natural resources, such as coal and oil, and contributes to the transition to electricity generation using natural and clean energy sources.
- Challenges of Solar Energy
- *Shading Effects*: partial shading significantly reduces PV output, may cause “hotspots” leading to long-term panel damage and can reduce the output of an entire module string.
- *High Temperature Effects*: increase cell temperature, results in lower voltage output.
- *Dust and Soiling Losses*: reduce solar radiation reaching the module surface and can decrease energy output by 5–25% depending on cleaning frequently.

b. Economic benefits of solar energy:

- *Cost Savings*: Solar systems can lead to substantial reductions in installation, maintenance, and operational costs over their lifespan.
- *Reduction in Fuel Costs*: Utilizing solar energy decreases the need for fossil fuels, minimizing recurring energy expenditures.

Contribution to Economic Growth:

- Investment in solar technology supports job creation and development within the renewable energy sector.
- *Energy Independence*: Solar adoption strengthens national energy security by reducing dependency on imported fuels and contributing to stable energy pricing.
- *Reduced Infrastructure and Transmission Costs*: distributed solar installations generate electricity near the point of use, reducing transmission and distribution losses.
- *Lower Environmental and Health Costs*: By reducing greenhouse gas emission and air pollutants, solar energy decreases health care costs related to pollution.
- Social Benefits of Solar Energy:

- *Energy Independence*: Solar energy helps achieve energy independence by reducing reliance on imported fuels.
- *Reduction in Electricity Bills*: Solar energy can significantly lower household electricity costs—homeowners in the UK have reported savings of up to 62% after installing solar panels^39^.

### Technical study

#### Designing electrical systems for buildings

The electrical load was calculated for each building model to estimate the cost of installing electrical systems and to evaluate the impact of using rooftop solar panels.

Estimation of Electrical Load:

The estimation considered an average electrical load of 8 kVA per 100 m^2^ of building area, in addition to other loads such as elevators and any other mechanical loads. The estimation is based on the Egyptian Electricity Regulatory Authority (March 2020) and Circular No. (4) for the year 2020 regarding design dispersion factors.

- Electrical load for Model A: 215 kVA
- Electrical load for Model B: 300 kVA

Description of Electrical Systems:

1. Electrical systems for buildings without using solar cells

Each building is powered through an internal electrical network, which consists of the main building panel and subsidiary panels for services and apartment panels, with connections extending to circuit breakers for the apartment panels.

2. Electrical systems with rooftops solar cells

In this case, the same electrical system used in the first case above is relied upon, with the addition of a solar energy panel for the building, which is installed on top of the building. The system is connected to each apartment’s panel and linked to the solar energy grid system (On Grid) through the EDMS 24–300, as per the requirements of the Holding Company for Electricity. Additionally, smart LED lighting is used for internal illumination in the buildings, along with smart meters.

The solar cells are designed to produce the maximum amount of solar energy and comply with the following standards and specifications for equipment:

- IEC 62,548, IEC 61,727, IEC 62,093, IEC 62,116, and IEC 62,109–1,2.
- NEC Articles 690 & 705.

Table 9 illustrates the electrical power generated by the solar cells for each building model based on the building area. A dedicated substation has been designed for each residential unit, and the power generated from the substation has been calculated as follows:

- 3 kW for substations used for buildings with areas of 490 m^2^ and 330 m^2^.
- 2 kW for substations used for buildings with areas of 180 m^2^ and 210 m^2^ in the 400 m^2^ model and the 330 m^2^ model.
- 2 kW for substations used for residential units of 150 m^2^ in the 330 m^2^ model and the 530 m^2^ model, as well as units of 100 m^2^ in the 530 m^2^ model.

The design of solar PV system will be under the concept of microgrid (MG) that is operating at grid-connected mode. Both of microgrid and utility grid will supply the demand of residential units. This will optimize the share of loading conditions, to not be at fixed concept of design and/or operating numbers and analysis. Another scale of flexibility in the design will be the consideration of diversity factor for loads, which reflects that not all loads within the units are working and connected at same timeslot. So, some rates of solar PV systems can supply different residential spaces.

From the data presented in Table 10 and the associated calculations, it is evident that the percentage of solar energy that can be provided from rooftop areas is approximately 29.7% of the required electrical load for Model A, and 28.9% for Model B.

3. Components of electrical systems with solar cells

**Table 10:** Electrical power generated by solar cells for each building model based on building area.

building type	Building area (m^2^)	Electrical load required per model (kVA)	Estimated solar cell apparent power (kVA)	Estimated solar cell active power (kW)
Model A	490	190.2	56.52	44.1
Model B	530	208.1	60.2	48

**Fig. 9:**
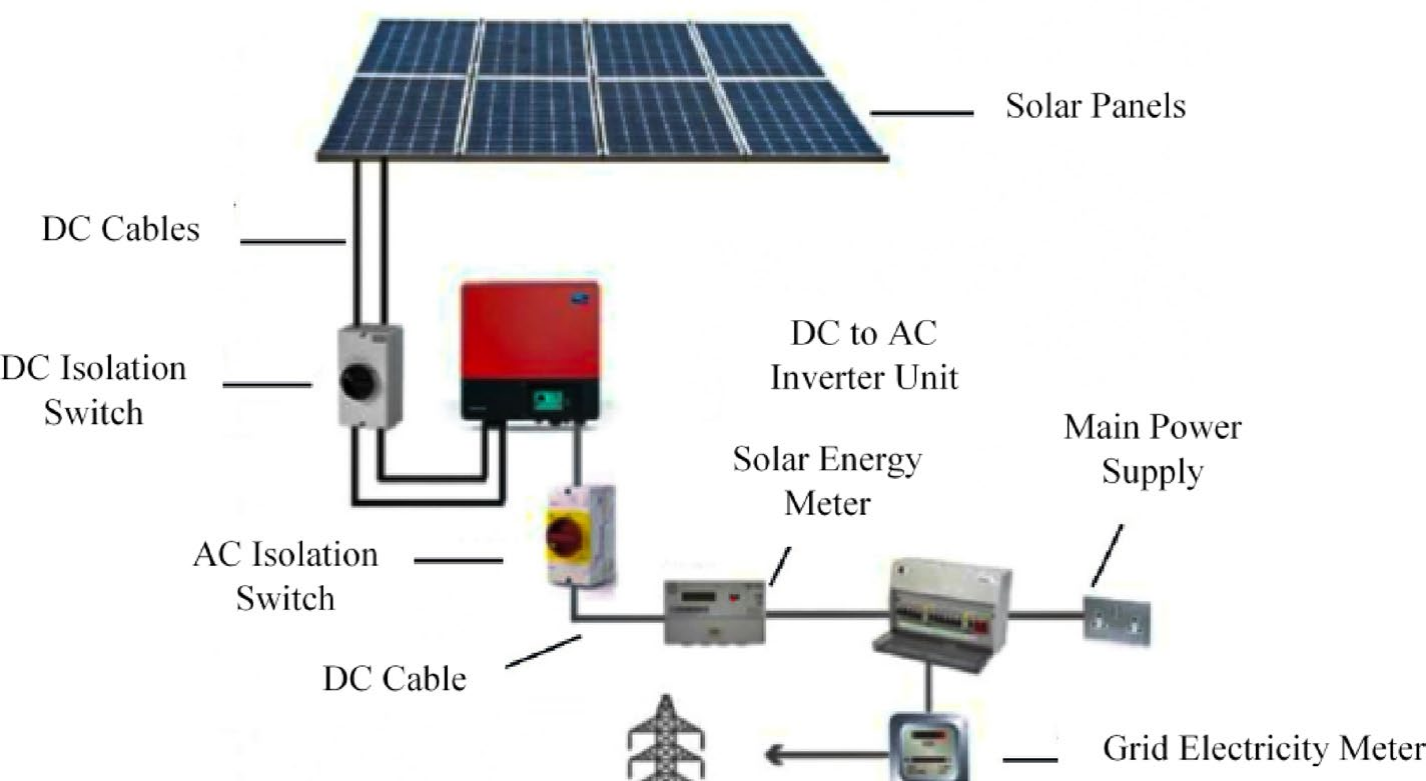
General diagram of the solar energy system.

The solar energy system consists of solar panels, DC cables, AC cables, an inverter to convert DC to AC, and a meter to calculate the consumed solar energy, as illustrated in Fig. 9.

**Fig. 10:**
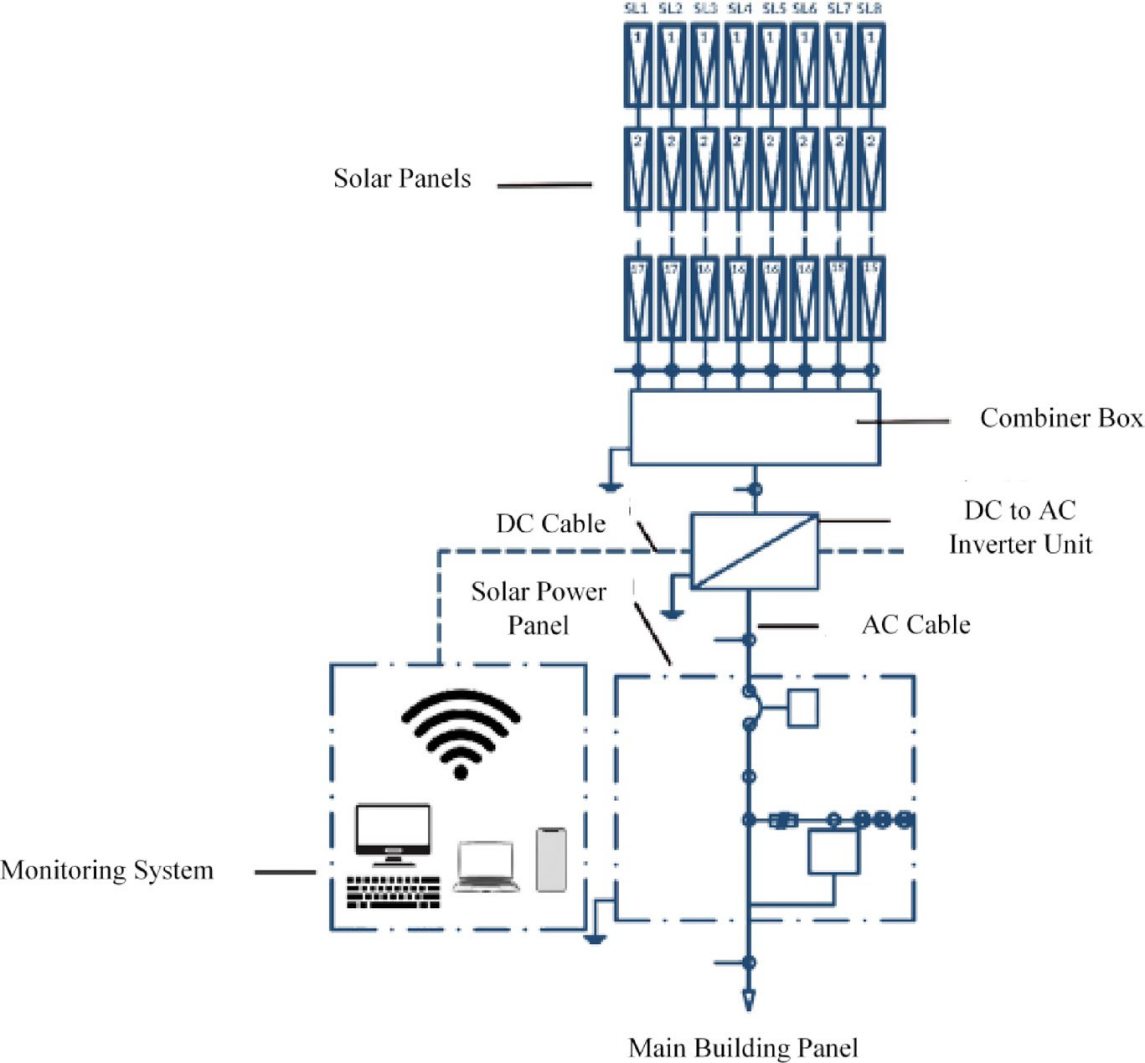
The connection of solar panels through the inverter to the main electrical panel.

Single-line diagram of the solar energy system for buildings:

This diagram (Fig. 10) illustrates the connection of solar panels through the inverter to the main electrical panel.

### Financial study of solar energy system

#### Cost in the case of a solar energy system

The cost in this alternative increases compared to the first alternative by the amounts shown in the Table 11, as it includes the initial cost of solar panels, the solar energy panels required for each building, and DC cables, while the total cost shown in Table 12.

**Table 11:** Cost in the case of a solar energy system.

Area (m^2^)	Building model	Electrical power from solar cells (kW)	Cost per kW (EGP)	Estimated initial cost for solar station per unit (EGP)	Estimated maintenance cost for the station over 10 years (EGP)
150	Model B: 530 m^2^	2	28,000	55,000	15,000
140	Model B: 330 m^2^	2	28,000	55,000	15,000
130	Model A: 490 m^2^ and Model B: 530 m^2^	2	,000	55,000	15,000
15	Model A: 490 m^2^	1.5	21,000	42,000	12,000
100	Model B: 530 m^2^	1.5	21,000	42,000	12,000

**Table 12:** Total cost in the case of a solar energy system.

Item	Surface Area (m^2^)	Electrical Capacity from Solar Cells (kW)	Cost per kW (EGP)	Cost of the Building in the Second Alternative (EGP)
Model A: 490 m^2^ Building	490	45	49,000	1,765,000
Model B: 530 m^2^ Building	530	51	42,500	2,050,000

The maintenance cost is approximately 2% of the initial cost, with a 5% annual increase as mentioned in the payback period calculations attached for each building model^37^.

The cost per kilowatt (kW) is as shown in Table 13.

**Table 13:** Cost per kilowatt of a solar energy system.

Item	Estimated Percentage of Total Cost (%)	Unit Price (EGP)*
Solar photovoltaic modules	38	15,200
Inverter	10	6,000
Installation	12	4,800
Cables and accessories	20	14,000
Total	100	40,000 per kW

*The prices are estimated and subject to change based on market rates.

##### Estimated cost of solar energy for 365 buildings

The total cost for using solar energy for 365 buildings is estimated to be approximately 666,455,000 Egyptian Pounds as calculated in Table 14.

**Table 14:** Estimated cost of solar energy for 365 buildings.

Item	Number of buildings	Cost per building (EGP)	Total cost (EGP)
Model A: 490 m^2^ building	287	1,765,000	506,555,000
Model B: 530 m^2^ building	78	2,050,000	159,900,000
Total initial cost			666,455,000

##### Return on investment

The return was studied over a 20-year period, and the findings indicated that the return on investment increases during the study period while the initial costs decrease. The payback period (Initial Payback Period) is approximately 10 years.

##### Annual electricity consumption calculation

The electricity consumption was calculated for two scenarios: Scenario 1 (consumption from the grid only) and Scenario 2 (consumption from the grid + alternative energy).

##### Scenario 1: grid-only consumption

In this case, all electricity demand is supplied entirely from the national grid.

- Assuming an average electricity consumption for a residential unit of 100 m^2^: Annual electricity consumption: 450 kWh, Annual electricity cost per unit: 6,162 EGP
- Assuming an average electricity consumption for a residential unit of 115 m^2^: Annual electricity consumption: 518 kWh, Annual electricity cost per unit: 7,088 EGP
- Assuming an average electricity consumption for a residential unit of 130 m^2^: Annual electricity consumption: 585 kWh, Annual electricity cost per unit: 8,012 EGP
- Assuming an average electricity consumption for a residential unit of 140 m^2^:Annual electricity consumption: 630 kWh, Annual electricity cost per unit: 8,650 EGP
- Assuming an average electricity consumption for a residential unit of 150 m^2^: Annual electricity consumption: 675 kWh, Annual electricity cost per unit: 9,245 EGP

##### Annual electricity costs for entire buildings

- Model A: Total annual electricity cost for all units in the building = 203,052 EGP
- Model B: Total annual electricity cost for all units in the building = 157,796 EGP

The total annual electricity bill cost for all residential apartment units amounts to: 14,497,528 EGP.

Characteristics: full dependence on grid electricity, high exposure to electricity tariff increases, no environmental benefit and no capital investment required.

##### Scenario 2: grid + alternative energy consumption

In this case, part of the electricity demand is covered by a solar energy system, reducing grid dependence.

- Assuming the average electricity consumption for a residential unit with an area of 100 m^2^ is 332 kWh, the annual electricity consumption cost per unit is 3,989 EGP.
- Assuming the average electricity consumption for a residential unit with an area of 115 m^2^ is 382 kWh, the annual electricity consumption cost per unit is 4,588 EGP.
- Assuming the average electricity consumption for a residential unit with an area of 180 m^2^ is 567 kWh, the annual electricity consumption cost per unit is 9,449 EGP.
- Assuming the average electricity consumption for a residential unit with an area of 210 m^2^ is 662 kWh, the annual electricity consumption cost per unit is 11,103 EGP.

##### Annual electricity costs for entire buildings

- The average annual electricity consumption cost for all residential units in Model A is 129,481 EGP.
- The average annual electricity consumption cost for all residential units in Model B is 100,884 EGP.

The annual electricity consumption cost for all project units is 9,148,979 EGP.

##### Technical & economic Interpretation between two scenarios:

*Scenario 1*: No carbon emission reduction, lower initial investment, higher long term operation cost and sensitive to future electricity price escalation.

*Scenario 2*: Reduce carbon footprint, reduces annual operating cost, improves long- term financial sustainability, provides partial energy independence.

Results of Using the Second Alternative:

Annual savings amount to 5,348,550 EGP, calculated as: 14,497,528—9,148,979 EGP.

## Sensitivity analysis: assessing economic and technical robustness

A screening sensitivity analysis (± 20%) was performed to examine how uncertainty in key assumptions influences (i) the potable-water offset achieved by supplying toilet flushing with treated greywater, (ii) the required treatment capacity (greywater generation), and (iii) the PV simple payback period. The use of ± 20% parameter variation as a sensitivity range is consistent with the approach reported for decentralized greywater reuse cost–benefit assessment, where multiple inputs are varied within − 20% to + 20%^40^.

Baseline values in this study were: population = 45,000 cap; greywater production Q_gw_ = 6,300 m^3^/day; toilet flushing demand Q_flushing_ = 2,700 m^3^/day; and baseline PV simple payback PB = 10 years. For toilet flushing only, the maximum potable-water offset is demand-limited by Q_flushing_. Accordingly, the baseline fraction of generated greywater required to fully supply toilet flushing is f_0_ = Q_flushing_ / Q_gw_ = 2700/6300 = 0.4286.

### Population and occupancy sensitivity (± 20%)

Occupancy was represented as a multiplicative factor on population, and therefore on both greywater generation (Q_gw_) and toilet flushing demand (Q_flushing_). Under this assumption, required treatment capacity scales with Q_gw_, while the maximum potable-water offset scales with Q_flushing_ (Table 15).

**Table 15:** Occupancy sensitivity (± 20%) and its effect on greywater generation, toilet flushing demand, and potable-water offset.

Scenario	Population (cap)	Q_gw (m^3^/day)	Q_flush (m^3^/day)	Max potable offset (m^3^/day)*	Max potable offset (m^3^/year)
Baseline	45,000	6,300	2,700	2,700	985,500
Occupancy − 20%	36,000	5,040	2,160	2,160	788,400
Occupancy + 20%	54,000	7,560	3,240	3,240	1,182,600

*For toilet flushing only, potable offset is demand-limited by Q_flushing_.

### Reuse fraction sensitivity (± 20%)

To isolate uncertainty in effective reuse, reuse was expressed as a fraction of generated greywater. Sensitivity was applied to the baseline fraction f_0_ as f = (1 ± 0.20)·f_0_. The treated and reused greywater volume supplied to toilet flushing was calculated as Q_reuse_ = min of (f·Q_gw_, Q_flush_) (Table 16).

**Table 16:** Sensitivity (± 20%) of the effective reuse fraction on treated greywater supply for toilet flushing and potable-water offset (baseline occupancy).

Scenario	Reuse fraction f	Treated & reused GW (m^3^/day)	Potable offset* (m^3^/day)	Potable offset (m^3^/year)
Baseline	0.4286	2,700	2,700	985,500
Reuse fraction − 20%	0.3429	2,160	2,160	788,400
Reuse fraction + 20%	0.5143	2,700	2,700	985,500

*For toilet flushing only, potable-water offset cannot exceed Q_flush_; therefore, increases in f above f_0_ do not increase potable-water savings unless additional end uses are included.

### Solar yield sensitivity (± 20%)

PV annual yield was varied by ± 20% as a screening assumption to reflect uncertainty in delivered energy (e.g., temperature effects, soiling, and shading). Simple payback is defined as the initial investment divided by annual savings. Let Y denote the annual PV energy yield (kWh/year) under the sensitivity case and Y_0_ the corresponding baseline annual PV energy yield (kWh/year). Assuming the electricity value per kWh is constant, annual savings are proportional to annual PV yield. Therefore, relative payback scales inversely with yield, giving PB_PV_ = PB_0_/(Y/Y_0_) for the ± 20% yield sensitivity (Table 17).

**Table 17:** PV yield sensitivity (± 20%) and its effect on PV simple payback.

Scenario	Yield factor (Y/Y0)	PV simple payback (years)
Baseline	1.00	10.00
PV yield − 20%	0.80	12.50
PV yield + 20%	1.20	8.33

## Results and discussion

Model results for the Janna residential compound indicate a greywater generation of 6,300 m^3^/day (2,299,500 m^3^/year) and, after applying a 5% backwash allowance, a hydraulic recovery of 95% (2,184,525 m^3^/year) (Table 1). Under the baseline end-use allocation, toilet flushing demand is 2,700 m^3^/day (985,500 m^3^/year); therefore, the achievable potable-water offset for toilet flushing is demand-limited by Q_flush_. The fraction of generated greywater required to fully satisfy flushing is f₀ = Q_flush_/Q_gw_ = 0.4286, and any additional recovered volume would require allocation to other non-potable end uses to translate into further potable-water savings. The ±20% screening confirms this behavior: occupancy variations scale both Q_gw_ and Q_flush_, while the effective reuse fraction affects delivered flushing supply, but potable-water savings remain capped at Q_flush_ for the toilet-flushing end use. For rooftop PV, the ±20% yield screening is evaluated using the inverse proportionality between annual yield and annual savings, giving PBPV = PB₀/(Y/Y₀), where Y is the annual PV energy yield (kWh/year) under the sensitivity case and Y₀ is the corresponding baseline annual yield.

The modeled greywater generation rate of 140 L/capita/day used in this study falls within the 80–160 L/capita/day range^21^ for middle- and high-income residential settings, and is consistent with the Egyptian Code assumption for luxury residential categories. The resulting CAPEX of 535 USD/(m^3^/day) is broadly consistent with published construction cost ranges of 160–300 USD/m^3^ for onsite greywater systems reported in the MENA region^34^, with the higher unit cost in this study reflecting the compound-scale dual-network infrastructure (in-building supply lines, collection networks, and a centralized treatment plant) rather than a simple building-level unit. The modeled OPEX of 0.06 USD/m^3^ is directly comparable to the 0.03–0.09 USD/m^3^ range reported for tertiary treatment facilities across MENA^34^ and within the 0.069–0.154 EUR/m^3^ range reported for Mediterranean reuse systems^35^, confirming that the cost assumptions adopted in this study are grounded in published operational evidence.

The LCOW of 0.133 USD/m^3^ on a treated-volume basis is competitive with published benchmarks for decentralized reuse systems. For comparison, Waris and Ghaith^17^ reported a PV-powered greywater treatment system for a 38-villa community in Dubai at significantly smaller scale, where unit costs are typically higher due to the absence of economies of scale. The compound-scale approach demonstrated in this study (365 buildings; ~45,000 residents) achieves a more favorable cost structure through shared infrastructure and centralized treatment, which is a key advantage of the integrated design concept proposed here. The surplus treated greywater volume beyond toilet flushing demand (approximately 1.20 million m^3^/year at baseline) represents a significant additional resource that could offset potable water use in landscape irrigation across the 89,670 m^2^ of green areas within the compound (Table 2), further improving the LCOW on an offset basis if these additional end uses are formally incorporated.

For the rooftop PV system, the solar coverage of approximately 29–30% of building electrical load (Table 9) is consistent with typical rooftop PV penetration rates in urban Egyptian residential buildings, where available roof area and shading constraints typically limit self-supply to 25–35% of total demand. The indicative simple payback of approximately 10 years is consistent with the broader trend of declining PV system costs documented by IRENA[45, 46], which reported a 90% reduction in the weighted-average levelized cost of solar PV electricity between 2010 and 2023 (from 0.460 to 0.044 USD/kWh). The ±20% PV yield sensitivity (payback range 8.33–12.50 years) reflects real-world uncertainty from dust and soiling losses of 5–25%, temperature derating, and inter-annual solar resource variability — all of which are particularly relevant to the hot, arid climate of New Cairo.

Taken together, the greywater and PV results demonstrate that an integrated resource-efficiency strategy at compound scale is both technically feasible and economically viable under the modeled conditions. The greywater system alone offsets 985,500 m^3^/year of potable water demand for toilet flushing — equivalent to 8.75% of the compound’s total annual potable water demand of approximately 11,250 m^3^/day. The PV system simultaneously reduces annual grid electricity costs by 5,348,550 EGP. This dual-resource approach directly supports Egypt’s national water security strategy and renewable energy targets, and provides a replicable, inventory-based framework that can be adapted to comparable large-scale residential developments across Egypt and the wider MENA region.

## Conclusion

This study provides a design‑stage assessment of integrating onsite greywater reuse with rooftop solar PV for a 365‑building residential compound (~ 45,000 residents) in New Cairo, Egypt.

### Key findings

- Greywater recovery and potable‑water offset (toilet flushing): Greywater generation is 6,300 m^3^/day (2,299,500 m^3^/year) and toilet flushing demand is 2,700 m^3^/day (985,500 m^3^/year). With a 5% backwash allowance, the assumed hydraulic recovery is 95% giving 2,184,525 m^3^/year recovered treated greywater.
- Greywater system costs: The total greywater system CAPEX is 3,370,000 USD, corresponding to 535 USD per (m^3^/day) of installed treatment capacity. The total OPEX is 0.0600 USD/m^3^ (annual total 137,970 USD/year at the design flow). Using the 20-year analysis horizon applied in the return assessment, the straight‑line levelized cost of water is 0.133 USD/m^3^ on a treated‑volume basis and 0.311 USD/m^3^ when expressed per m^3^ of potable‑water offset for toilet flushing only (conservative, demand‑limited).
- Rooftop PV economics (as reported): The estimated total PV initial cost for 365 buildings is 666,455,000 EGP. Based on the reported Scenario 1 vs Scenario 2 electricity costs, the modeled annual electricity cost saving is 5,348,550 EGP, giving an indicative simple payback of ≈10 years. A ± 20% PV‑yield screening gives a payback range of 8.33–12.50 years.

### Recommendations for future research and implementation

- Report PV electricity generation outputs (monthly and annual) and the associated performance assumptions used in the model.
- Establish an operational monitoring plan (flow and treated‑water quality) to verify recovery, treatment performance, and compliance for the intended reuse.

## Supplementary Information

Below is the link to the electronic supplementary material.

Supplementary Material 1

## Data Availability

Data is provided within the supplementary information files.
