# Supplementary material for: Greywater recycling and solar photovoltaic integration for sustainable water and energy management in urban Egypt
Source: Sci Rep. 2026 May 6;16:14389. doi: 10.1038/s41598-026-49932-y (PMC13149641; doi:10.1038/s41598-026-49932-y)
Supplement: Supplementary file 1 — Supplementary Material 1 [file 41598_2026_49932_MOESM1_ESM.pdf]

Ministry of Housing, Utilities & Urban Communities

New Urban Communities Authority

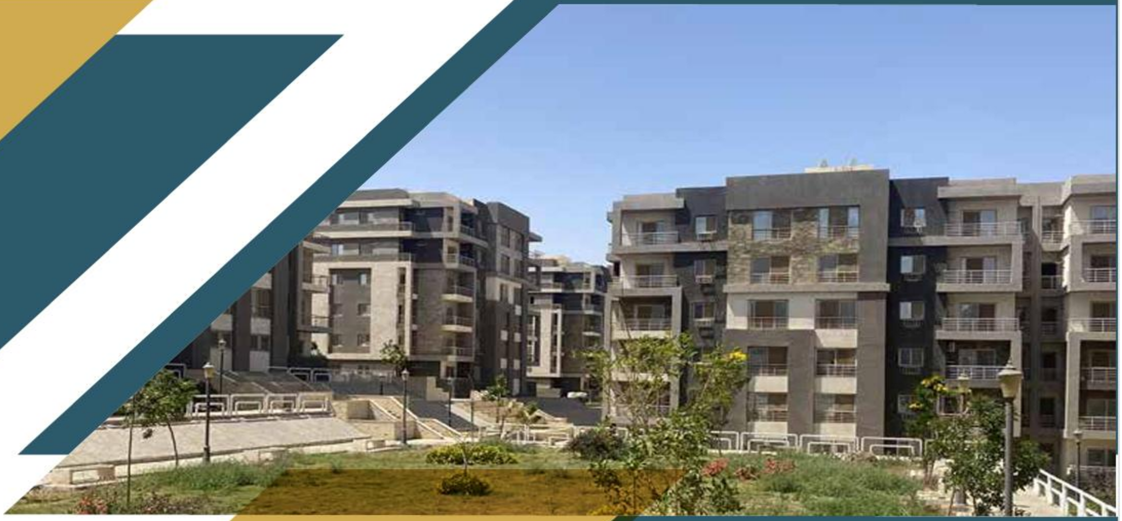

Booklet of Terms and Conditions for Reserving Residential Units

For allocation through a public lottery system via the website: https://reserve.newcities.gov.eg

Number of units: 782 residential units

Number of cities: 3 cities

Project: Jannah (Second Offering)

Registration start date: Sunday, 27/10/2024

Cities Included in the Offering

- New Cairo (409 units)
- New Damietta (150 units)
- New 6th of October (223 units)

  Including 5% of units allocated for people with disabilities

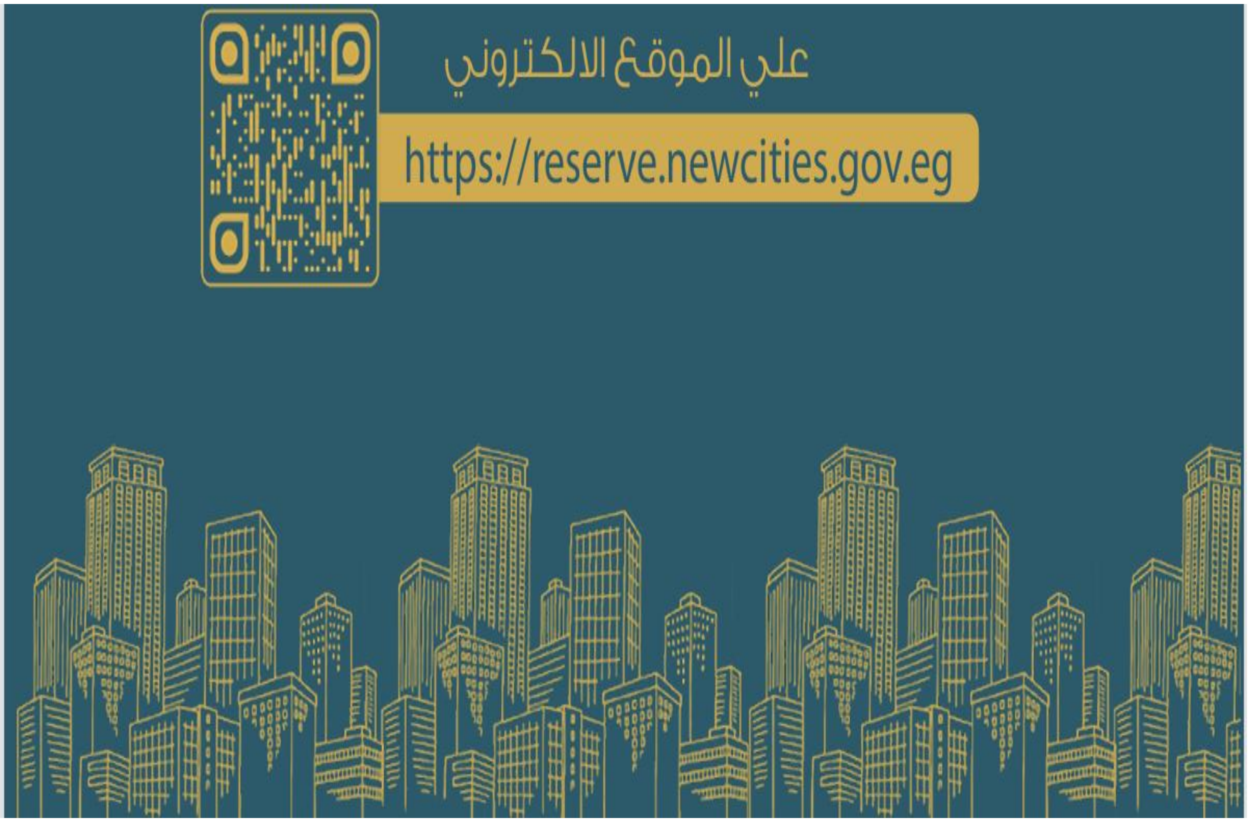

Contents

- Introduction
- Reservation terms and general conditions
- Steps for online reservation
- Financial conditions
- Real estate conditions
- Cases and rules for cancellation of allocation
- Technical notes
- Floor plans

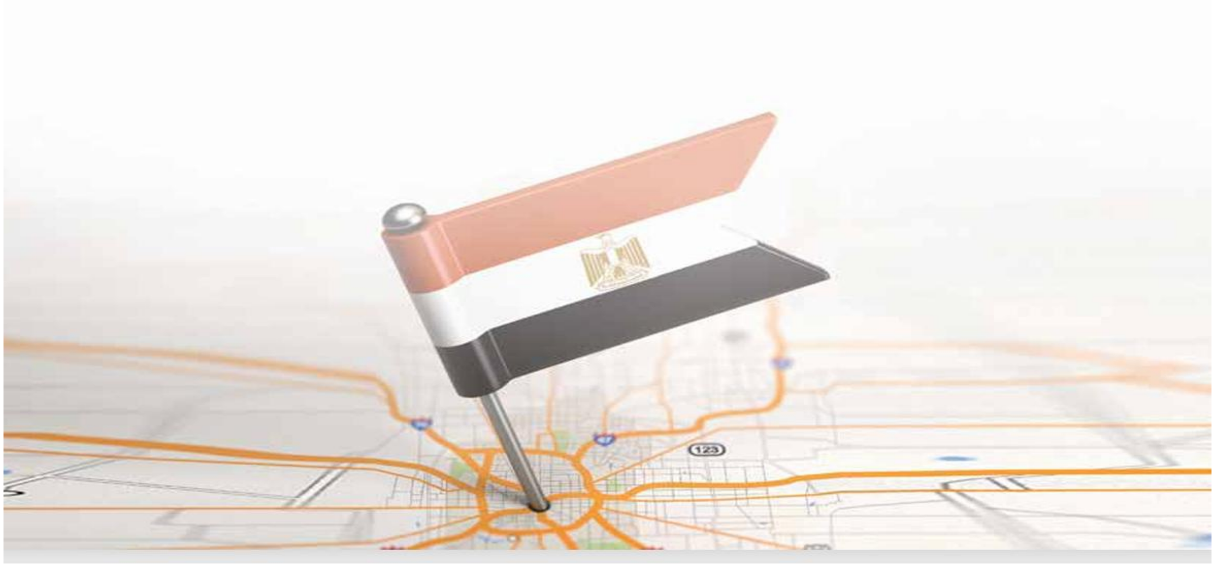

Introduction

The New Urban Communities Authority was established under Law No. 59 of 1979, with the aim of developing new urban cities and communities in desert areas. This is intended to reduce population pressure on the Nile Valley and Delta and to expand beyond the limited existing urban boundaries.

Since its establishment, the Authority has been working to develop modern communities according to the latest urban planning and architectural standards, ensuring a healthy environment and suitable housing for citizens.

As part of the Ministry of Housing, Utilities, and Urban Communities' efforts to provide adequate housing for different segments of society, the Authority is offering fully finished residential units under the **Jannah Project**.

This offering includes:

- 782 residential units
- Across 3 cities
- Unit areas ranging from 100 m² to 150 m²

Including 5% of units reserved for people with disabilities

Reservation Conditions

1. The applicant must be a **natural person of Egyptian nationality** (not a legal entity).
2. The applicant must be **at least 21 years old** at the time of the announcement and have full legal capacity to contract.
3. A household (**husband and wife**) is **not allowed to reserve more than one unit**, except for people with disabilities.
4. People with disabilities, or their legal guardians, are allowed to apply for reservation.
5. This (announcement) and the **booklet of terms and conditions** are considered an **integral part of the sale contract** and complementary to its provisions.
6. The provisions of:
   - Law No. 59 of 1979 (New Urban Communities)
   - Law No. 119 of 2008 and its executive regulations
   - The real estate regulations of the Authority

shall apply and complement any matters not explicitly stated in this booklet.

Reservation Conditions

7. The residential units offered are **strictly for residential use only**.
   The buyer (or any successor) is **not permitted to change the designated use** of the unit.
   In case of violation, the Authority has the right to:
   - Cancel the contract without prior notice
   - Take all necessary legal actions in accordance with applicable regulations
     (Note: Unit prices are set based on residential use only.)
8. The applicant acknowledges that they have **reviewed and accepted all terms, specifications, and information** provided.
   Submitting the application and paying the reservation deposit constitutes **final approval** of these terms.
9. Applying for any unit is considered **conclusive evidence** that the applicant:
   - Accepts all terms stated in the booklet
   - Has inspected the site and is fully aware of its conditions

10. Any matter not specifically addressed in these conditions shall be subject to the **rules, regulations, and decisions** of the New Urban Communities Authority and its affiliated bodies, without objection from the applicant now or in the future.

11. Each residential unit includes a **proportional share of the land**, calculated based on the unit's area relative to the total building area.

Special Procedures for People with Disabilities

- New Cairo (20 units)
- New Damietta (7 units)
- New 6th of October (11 units)

- Applicants (or their guardians) must register on the website and provide:
  - Name
  - National ID number
  - Email
  - Phone number
  - Address
  - Integrated Services Card number
- Required documents:
  - Copy of National ID
  - Integrated Services Card
- Applicants must:
  - Select the desired city and project
  - Choose the floor (ground / typical / last)

- Pay the reservation deposit and study fees using the provided payment methods
- Upload proof of payment

- After the application period ends:
  - Applications are reviewed
  - Eligible applicants are notified of the **date and location of the lottery**
- The lottery system is conducted:
  - First for people with disabilities (within the 5% quota)
  - Then for the applicants
- The relevant city authority verifies the validity of documents before proceeding with contracting.

Reservation Steps for Residential Units (Immediate Booking)

Application Period:
From 27/10/2024 to 26/12/2024

Applicants must access the website:
https://reserve.newcities.gov.eg
and follow these steps:

Step 1: Registration

1. Review the **terms and conditions booklet** carefully and ensure all required data is prepared before applying (written/video guides are available).
2. Create a **new account** (if not previously registered) by entering:
   - National ID number and full name (as per ID)
   - Email address (**mandatory** – main communication method)
   - Mobile number
   - Governorate and current address (**mandatory**)
3. Enter additional details:
   - Marital status
   - Spouse's name and national ID (if applicable)
4. Upload a **copy of the applicant's national ID** (front and back in one file).

5. Create and confirm:
   - Password
   - Verification code
6. Activate the account via the link sent to the registered email.
7. Log in using your email and password.

Step 2: Submitting the Reservation Request

1. Browse available units under the housing section and select:
   - City
   - Project
   - Floor (ground / typical / last)
2. Upload required documents (in one PDF file, max 2 MB), such as:
   - Spouse documents (if applicable)
   - Birth certificates for minor children
3. Submit the reservation request.
   A message will confirm that the **initial application has been saved**.

⚠️ Note: The reservation is **not final** until payment is completed.

Step 3: Payment

Applicants must pay:

- **Reservation deposit (**seriousness fee**)**
- **Application study fee** (non-refundable)

Available Payment Methods:

1. Fawry Payment

- Use the reference number to pay via:
  - Fawry outlets
  - Fawry Plus
  - Mobile apps / ATMs / bank services

2. Bank Card

- Enter card details (number, expiry date, CVV)
- Complete verification through your bank (OTP)

3. Bank Transfer

- Transfer to Housing and Development Bank (HDB)
- Use SWIFT code: **HDBKEGCAXXX**
- Include the **18-digit reservation code**

After payment:

- The system verifies the transaction
- You will receive an **SMS confirmation**
- Upload proof of payment (PDF / JPG / JPEG)
- Print and sign the reservation form, then upload it

Important Note

- **Deadline for payment:** 26/12/2024 at 2:00 PM

Step 4: Lottery Process

- Applications are reviewed by the Authority
- A **public lottery** is conducted among applicants based on unit types (ground / typical / last floor)
- Applicants are notified via:
  - Email
  - SMS

Step 5: Post-Lottery

If allocated a unit, the applicant must:

- Visit the relevant city authority
- Pay:
  - 20% of unit price
  - 1% administration fees
  - 0.5% Board of Trustees fee

  Within **one month from the lottery date**

1. Governorate and current address (**mandatory**)
2. Enter additional details:
   - Marital status
   - Spouse's name and national ID (if applicable)

3. Upload a **copy of the applicant's national ID** (front and back in one file).
4. Create and confirm:
   - Password
   - Verification code
5. Activate the account via the link sent to the registered email.
6. Log in using your email and password.

Submitting the Reservation Request

1. Browse available units under the housing section and select:
   - City
   - Project
   - Floor (ground / typical / last)

1. Upload required documents (in one PDF file, max 2 MB), such as:
   - Spouse documents (if applicable)
   - Birth certificates for minor children
2. Submit the reservation request.
   A message will confirm that the **initial application has been saved**.

⚠️ Note: The reservation is **not final** until payment is completed.

Important Note

- **Deadline for payment:** 26/12/2024 at 2:00 PM

Step 6: Refund (If Not Allocated)

- **Fawry:** Refund via Fawry outlets (within one month)
- **Bank card:** Refund to the same card
- **Bank transfer:** Refund to the same account (minus bank fees)

Financial Conditions

First: Unit Prices

- Unit prices are determined **per square meter**, as shown in the attached unit tables.
- Prices include **distinction percentages** (location/floor advantages) for each unit.

Payment Method

1. Upon Contracting

- A **maintenance deposit** of **8% of the unit price** must be paid at the time of contracting (as detailed in the real estate conditions).
- An **advance maintenance amount** of **1% of the unit price** is paid for the first year from the date of delivery (under account), until

returns from the maintenance deposit are generated. Annual financial settlement will be conducted thereafter.

- Additional costs to be paid:
  - Contracting fees (as per regulations)
  - Utility connection fees (gas, electricity, water), when provided

---

2. Reservation Payment

- A **reservation deposit of EGP 100,000** must be paid.
- An additional **EGP 500** is paid as a **non-refundable application study fee**.

Payment must be made in **one installment** through one of the following methods: (Fawry, Bank card and Bank transfer)

3. Payment of Remaining Unit Value

Installment System

- The applicant must:
  - Pay **20% of the total unit price** (plus administrative and trustee fees) within one month after the lottery
  - Pay 10% upon unit delivery
- The remaining 70% of the unit price is paid in installments as follows:
  - Equal quarterly installments over 3, 5, or 7 years
- Installments are subject to:

- Interest based on the Central Bank of Egypt rate at the time of due payment (**2% (Ministry of Finance regulation and 0.5% collection fee)**

- The **first installment** is due **3 months after unit delivery**, and post-dated checks must be submitted before delivery.

4. Early Payment Option

- If the buyer wishes to:
    - Pay the full amount
    - Or pay more than the required 30% upfront

Real Estate Conditions:

1. Ownership and Transfer Restrictions

- The allocation of the unit is **personal** and **may not be transferred, assigned, or disposed of** in any way (whether fully or partially) without:
    - Prior **written approval** from the Authority
    - Full payment of all dues up to the date of transfer request
    - Payment of all prescribed administrative fees
- All procedures must comply with the **real estate regulations** of the Authority.

---

2. Cases of Cancellation of Allocation

The allocation of the unit may be cancelled in the following cases:

- Violation of any **reservation conditions or provisions** stated in the booklet
- Submission of a **cancellation request by the beneficiary**
- Failure to pay the required **20% + (1% administrative + 0.5% trustee fees)** within the specified timeframe
- Failure to pay the required **20% + (1% administrative + 0.5% trustee fees)** within the specified timeframe
- Failure to pay **two consecutive installments**
- Disposing of or transferring the unit **without prior approval** from the Authority or the relevant city authority
- Changing the **designated use** of the unit (non-residential use)
- Failure to **contract or receive the unit** within the specified timeframe

---

3. Financial Deductions in Case of Cancellation (Before Delivery)

If the allocation is cancelled **before receiving the unit**, the following will be deducted:

- **1% administrative fees**
- **0.5% Board of Trustees fee**
- These deductions are calculated from the **total unit price**

4. Financial Deductions in Case of Cancellation (After Delivery)

If cancellation occurs **after receiving the unit**, the following will be deducted:

- **1% administrative fees**
- **0.5% Board of Trustees fee**
- **7% annual occupancy fee** from the total unit price
  - Calculated from the **date of receiving the unit until its return**

---

5. Maintenance Obligations (Owners Association)

- In accordance with Law No. 119 of 2008 and its executive regulations:
  - The unit owner must pay a **maintenance deposit of 8% of the unit value** at contracting, allocated to:
    - Owners' association
    - Or a management company responsible for maintenance
- This fund is used for:
  - Security
  - Cleaning
  - Maintenance of project facilities
  - This is a **mandatory condition for contracting**.
  - A financial settlement is conducted **annually**, and the owner must pay any additional maintenance costs exceeding the deposit returns.
  - Maintenance costs vary depending on:

- Unit size
- Floor
- Other factors
- An additional **1% estimated maintenance cost** is paid in advance for the first year (under account), with settlement at year-end.

Official Correspondence:

- All official communications issued by the Authority or city authority:
  - Are considered **legally valid and binding**
  - As long as they are sent to the address registered in the reservation application
  - **Technical Conditions**
  - The **unit area** includes:
  - Wall thickness
  - The unit's proportional share of **common areas**, such as:
  - Entrances
  - Staircases
  - Courtyards
  - Other shared facilities
  -
  - The buyer is **not allowed to make any modifications** to the unit without:

- Referring to the relevant city authority
- Obtaining official approval from both:
- The Authority
- The city authority
- Applying for any unit is considered **conclusive proof** that the applicant:
- Has fully inspected the site and unit
- Is fully aware of all details, layouts, and specifications
- Has no claims of lack of knowledge
- ---
- The **distinction percentages** (location/floor advantages) listed in the unit tables are **final**.
  However, if upon delivery the Authority determines additional distinction value, the buyer is obligated to **pay the difference**.
- ---
- **Important Notes**
- Unit sizes, layouts, and specifications shown in the booklet are binding.
- The Authority reserves the right to apply all applicable laws and regulations in any situation not explicitly covered.
- The project includes:
- Fully finished residential units
- Shared facilities and services managed collectively

- Buyers must comply with:
- All financial obligations
- Maintenance requirements
- Legal and administrative procedures
-

By applying for a unit, the applicant confirms:

- Full acceptance of all **terms and conditions**
- Commitment to **payment schedules and fees**
- Understanding of **legal restrictions (no resale without approval)**
- Awareness of **penalties in case of cancellation or delay**

Model A

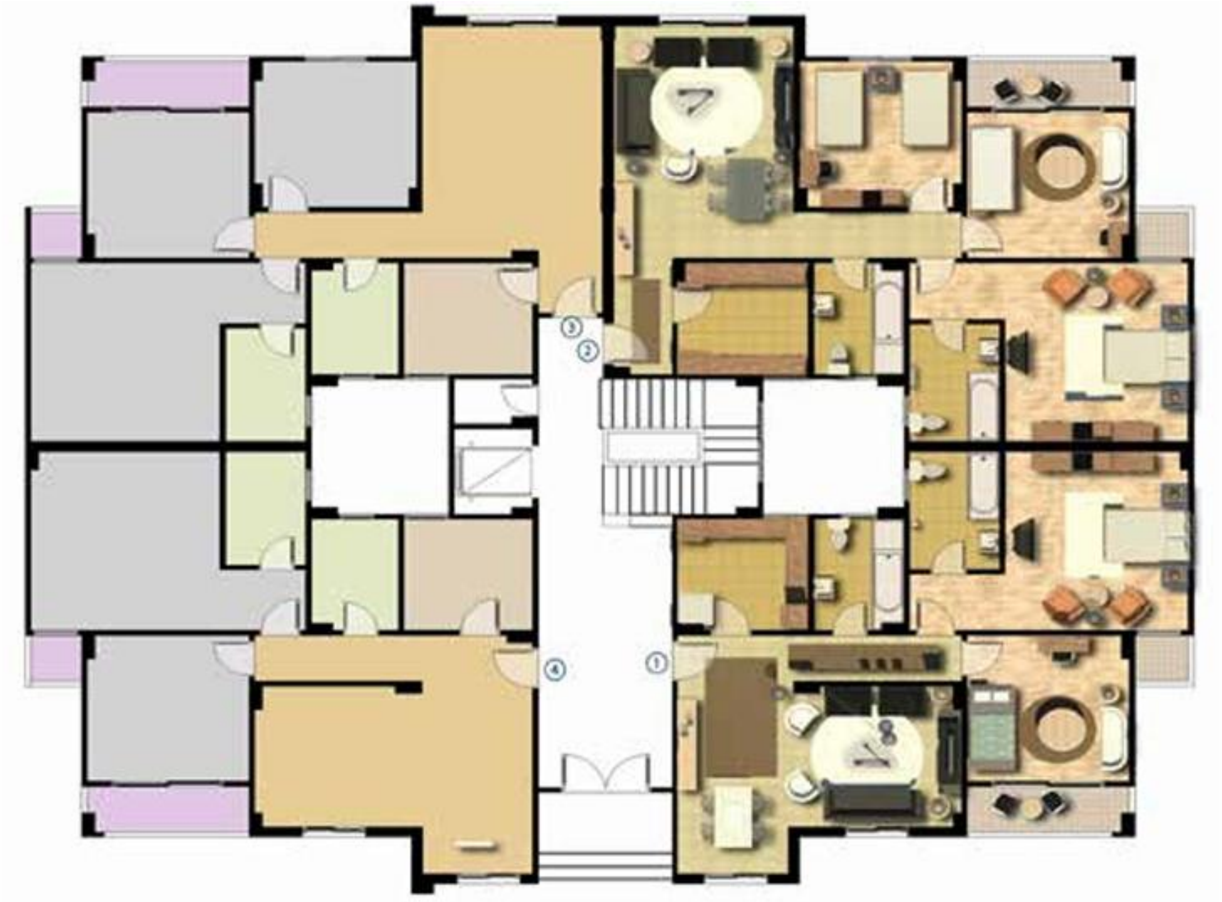

Ground Floor plan

Unit Number	Area, m^2
1	115
2	130
3	130
4	115

Model A

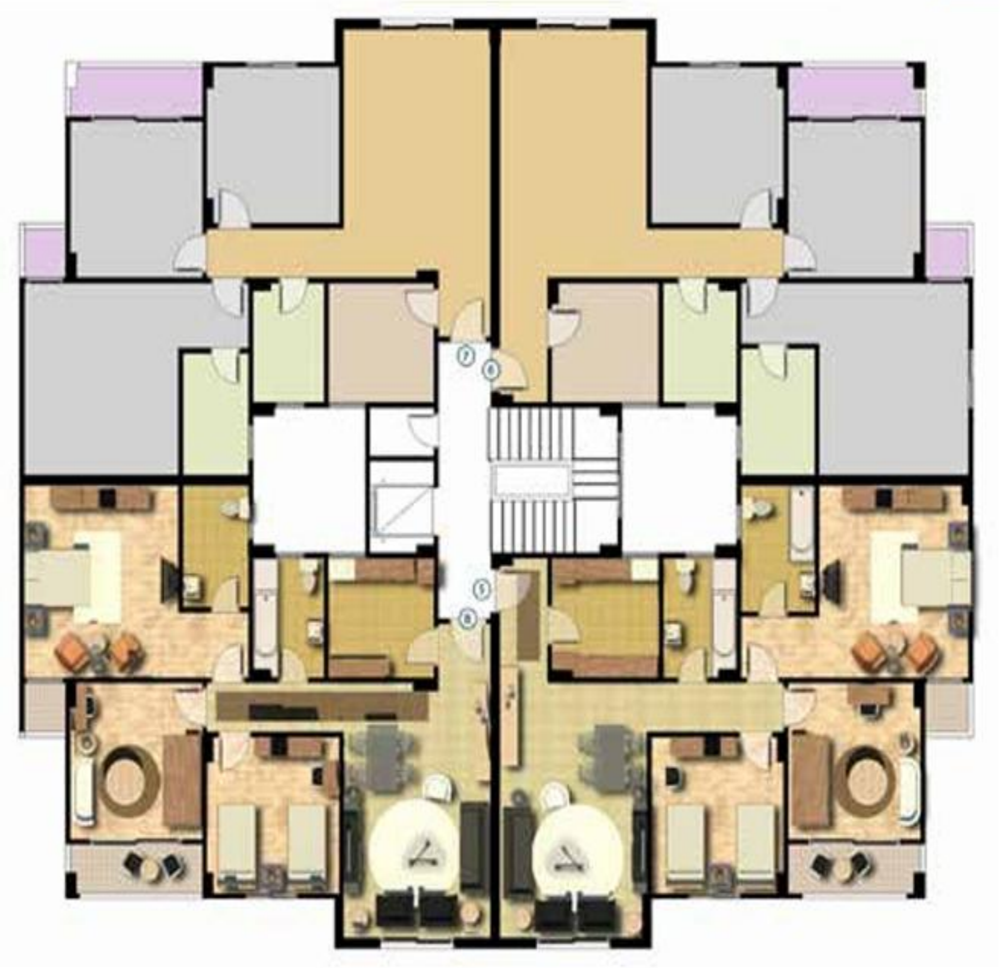

Typical Floor plan

Unit Number	Area, m^2
5	130
6	130
7	130
8	130

Model B

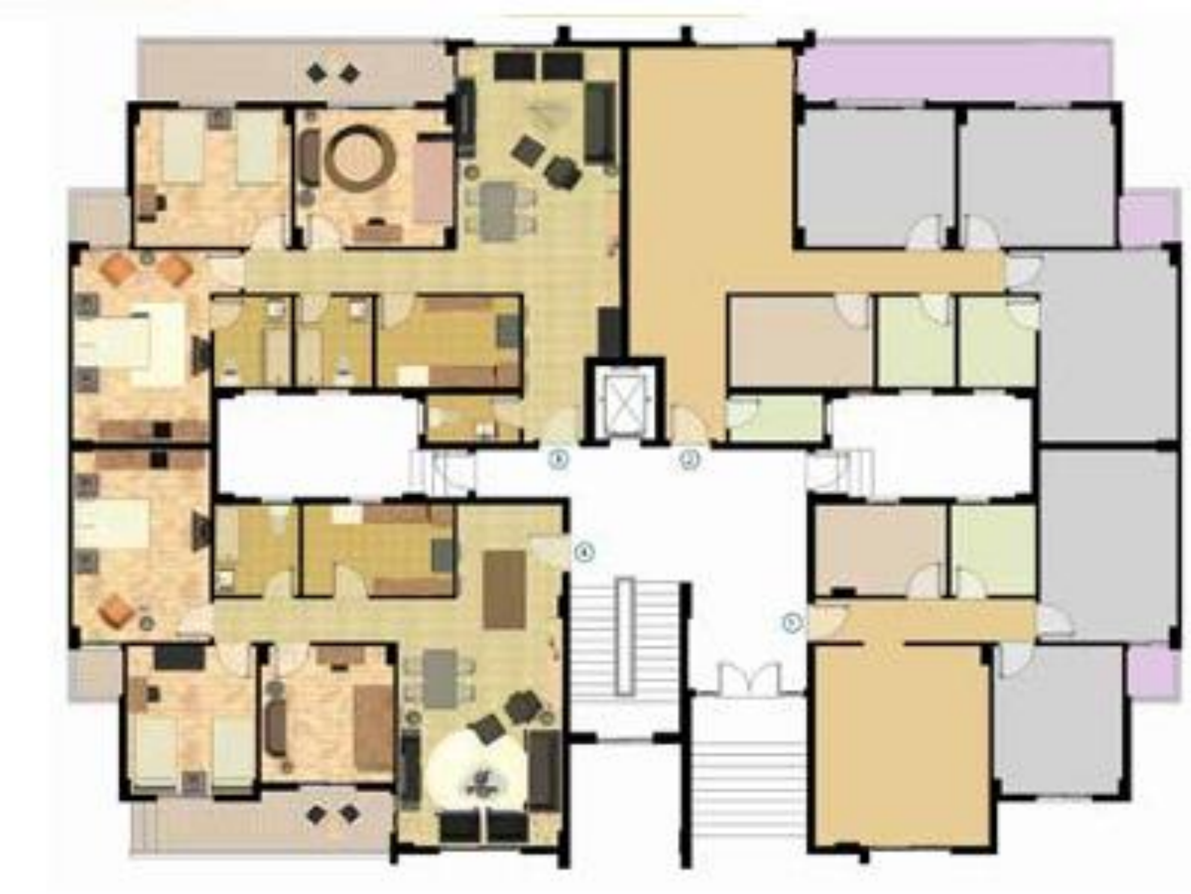

Ground Floor plan

Unit Number	Area, m^2
1	115
2	130
3	130
4	115

Model B

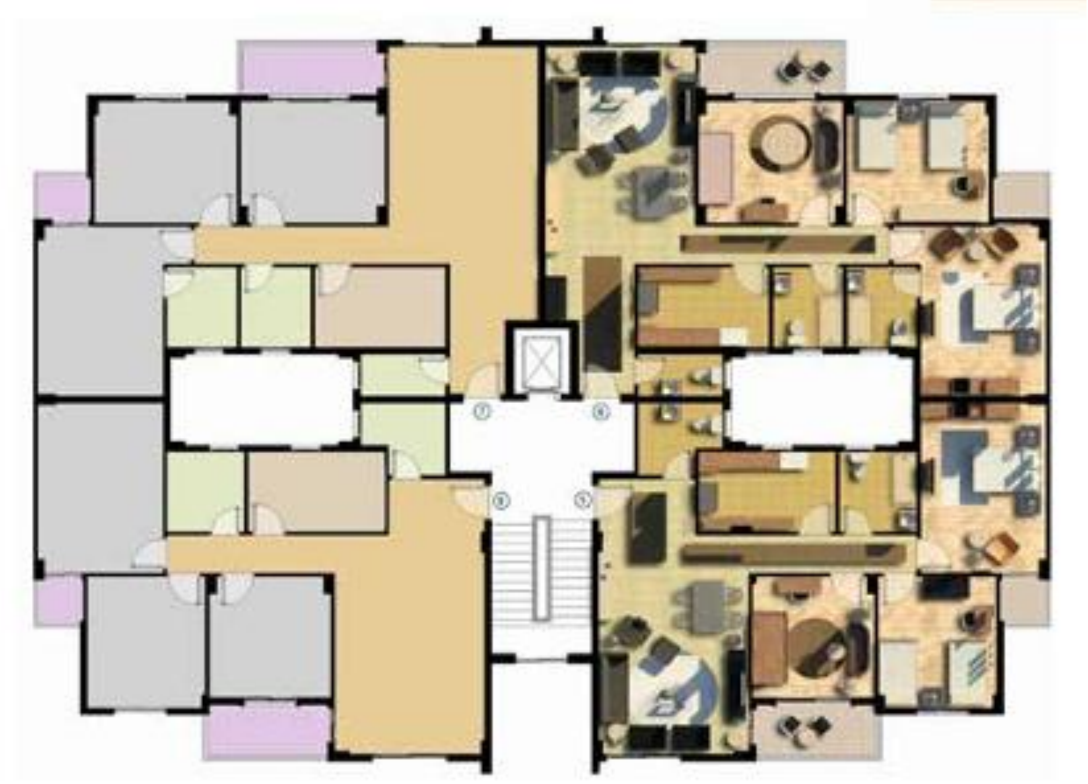

Typical Floor plan

Unit Number	Area, m^2
5	130
6	130
7	130
8	130
